# Person-Message Fit: Racial Identification Moderates the Benefits of Multicultural and Colorblind Diversity Approaches

**DOI:** 10.1177/0146167220948707

**Published:** 2020-09-15

**Authors:** Teri A. Kirby, Cheryl R. Kaiser

**Affiliations:** 1 University of Exeter, UK; 2 University of Washington, Seattle, USA

**Keywords:** racial identity, self/identity, prejudice/stereotyping, organizational behavior, intergroup processes, multicultural, colorblind, diversity, inclusion, self-stereotyping

## Abstract

Although diversity approaches attempt to foster inclusion, one size may not fit all. In five studies, African Americans (*N* = 1,316), who varied in strength of racial identification, contemplated interviewing at a company with a multicultural or colorblind approach. Participants in the multicultural condition anticipated pressure to be prototypical group members relative to colorblind and control conditions. Only weakly identified participants reacted to this pressure, experiencing more anxiety and inauthenticity in the multicultural relative to colorblind (not control) company. Strongly identified participants experienced *less* anxiety and inauthenticity in the multicultural relative to colorblind and control companies. Inauthenticity among weakly identified participants was apparent in self-descriptions and linked with worse hiring outcomes in multicultural relative to colorblind and control contexts. Despite predictions, there were no self-stereotyping effects. Diversity approaches that make some group members more comfortable may prove simultaneously constraining for others, highlighting the complexity in how diversity approaches affect individuals.

Despite long-standing efforts to reduce employment discrimination in the United States, racial minorities continue to be underrepresented and report feeling unwelcome in many workplaces (Bureau of Labor Statistics, 2019; Sinclair & Kunda, 1999; Steele et al., 2002; U.S. Census Bureau, 2012). To create a more welcoming climate, many companies implement diversity initiatives or statements affirming the importance of group differences (Dobbin, 2009), also known as a multicultural approach. For example, Bank of America (2014) notes, “At Bank of America, we realize the power of our people and value our differences—in thought, style, sexual orientation, gender identity, culture, ethnicity and experience—recognizing that our diversity makes us a stronger company.” Although this approach to diversity may create a welcoming climate, highlighting group differences could prove problematic for some racial minorities. For instance, Erica Baker Joy (2014), a former Google employee, documented her experience navigating workplace expectations in a blog post:

I am constantly making micro-evaluations about whether or not my actions will be attributed to my being “different” . . . I have to navigate the expectation of stereotypical behavior and disappointment when it doesn’t happen (e.g. my not being the “sassy black woman”).

Indeed, when a group is underrepresented in a particular context, members of that group often grapple with the expectation that they will serve as a representative or prototype of their group (Bell & Nkomo, 2003; Kanter, 1977; Sekaquaptewa et al., 2007). This is particularly the case in work contexts, where there are strong impression management demands. Because cues in the environment can send messages about expected behavior (Sinclair et al., 2005), we suggest that *diversity approaches*, such as multiculturalism and colorblindness, may also send cues about how racial minorities in particular should present themselves. The current research explores how the fit between organizational diversity approaches and individual differences in racial identification interacts to predict authenticity and related outcomes among racial minorities. As environmental fit is central to motivation and the self (Schmader & Sedikides, 2018), understanding the implications of organizational approaches to diversity for different individuals can provide significant insights into the experiences of underrepresented groups.

## Diversity Approaches

Diversity approaches (or philosophies, ideologies, or strategies) are sets of ideas about how people from different backgrounds should interact, relate, and accommodate each other (Plaut, 2002). These approaches come in many forms, but two of the most prominent and well-understood approaches are multiculturalism and colorblindness (Gündemir et al., 2019; Plaut, 2002). Multiculturalism highlights racial and ethnic differences, arguing that these differences enrich society and should be celebrated (Hahn et al., 2015; Plaut, 2002). Colorblindness^1^ instead deemphasizes differences, focusing on individual traits or similarities across people, considering this commonality a source of strength.^2^

The dominant social psychological narrative contends that multiculturalism imparts important psychological benefits to minorities relative to colorblindness (e.g., Purdie-Vaughns & Walton, 2011). Not only do minorities prefer multiculturalism over colorblindness (Ryan et al., 2007), but organizational multicultural approaches also facilitate engagement and trust among minority employees (Plaut et al., 2009; Purdie-Vaughns et al., 2008, but see Wilton et al., 2020). For example, African Americans contemplating employment at a racially homogeneous company experience fewer identity-related concerns and higher trust when the company has a multicultural as opposed to a colorblind recruitment brochure (Purdie-Vaughns et al., 2008). Similarly, the more White employees at companies endorse multiculturalism, the more minorities in those companies are psychologically engaged in their work (Plaut et al., 2009), and the reverse is true for colorblindness. Because minorities have chronic and well-justified concerns about belonging in domains where they have been historically devalued, instilling a sense of belonging in these environments can help overcome these barriers (Steele et al., 2002).

## The Impact of Diversity Approaches on State Authenticity

The benefits of multiculturalism dovetail with a broader research literature in the social identity threat tradition showing that many cues in an environment, such as representation of members of one’s group, can signal belonging and fit (Kirby, Tabak, et al., 2020; Murphy et al., 2007; Steele et al., 2002; Walton & Cohen, 2007), as well as reduce concerns about discrimination (Brady et al., 2015; Dover et al., 2014; Kaiser et al., 2013; Kirby et al., 2015). Although the importance of belonging has received a great deal of attention (see Baumeister & Leary, 1995), state authenticity has rarely been examined as a component in facilitating inclusive workplaces, despite being theorized as distinct from belonging and related outcomes (Schmader & Sedikides, 2018). State authenticity is “the sense or feeling that one is currently in alignment with one’s true or genuine self; that one is being their real self” (Sedikides et al., 2017, p. 521). People who feel authentic at work experience increased well-being, work engagement, job satisfaction, and performance (Ménard & Brunet, 2011; Metin et al., 2016). To achieve state authenticity, one’s sense of “fit,” or the matching of characteristics of the environment with internal characteristics of the self, may be crucial (Schmader & Sedikides, 2018).

Because racial minorities experience less fit in majority White environments (Schmader & Sedikides, 2018), one might expect minorities to feel more authentic in a multicultural than colorblind context. However, authenticity depends on the fit between organizational and personal values or norms (i.e., “goal fit”). Because multiculturalism and colorblindness prescribe different models for navigating diversity, the appeal of these approaches may depend on a particular person’s values.

In particular, individuals’ level of group identification may be critical in understanding the experience of authenticity in multicultural and colorblind contexts. Although group identification consists of several dimensions (Leach et al., 2008), we focus on centrality, or the extent to which a particular group membership is chronically central to one’s sense of self (Leach et al., 2008), because it is relatively stable rather than responsive to situational context (Major et al., 2002). In line with social identity theory (Tajfel & Turner, 1986), the self can involve a range of identities, including both the individual (or personal) self and group (or collective) identities. Whereas strongly identified group members tend to prioritize their group identity, those weakly identified are less comfortable prioritizing the group^3^ and are more likely to embrace the individual self. This latter point is particularly true if their group is underrepresented or low status (Barreto & Ellemers, 2000; Ellemers et al., 2002; Spears et al., 1997).

For instance, when women’s devalued group identity is made salient, those who are weakly identified with their gender group display pro-male biases (Brady et al., 2015; Derks et al., 2011), a reaction that might suggest discomfort with the focus on their identity. In addition, people report reduced comfort, well-being, and authenticity, as well as increased identity-based anxiety, when imagining or being in environments that are incompatible with their group identity or orientation (Knight & Haslam, 2010; Ng et al., 2020; Schmader & Sedikides, 2018). Accordingly, international students who are less oriented toward their home culture report less comfort and a weaker sense of belonging in their surroundings when completing a test in a space designated specifically for international students (Ng et al., 2020). Conversely, international students who are more oriented toward their home culture report more comfort and belonging when completing a test in an international student space.

These findings suggest that environments that make group identity salient (or focus on group differences, as in the case of multiculturalism) may not be a good fit for those who are weakly identified with the group, thus reducing authenticity. Weakly identified group members might instead feel more authentic in environments with a colorblind approach, as this approach downplays group identity. In contrast, those strongly identified with their group may find multiculturalism compatible with their sense of self, increasing authenticity at work, but may find colorblindness to be less compatible. In addition, this lack of fit may translate to increased state anxiety—that is, “a transitory emotion characterized by physiological arousal and consciously perceived feelings of apprehension, dread, and tension” (Fan & Shi, 2009, p. 67); see also Endler & Kocovski (2001); Spielberger (1966)—as trait inauthenticity is associated with negative well-being outcomes (Kernis & Goldman, 2005; Sheldon et al., 1997; Wood et al., 2008).

## The Role of Group Identification on Impression Management Behaviors

Diversity approaches that are incompatible with one’s sense of self (an individual or a group identity focus) may further lead to pressure to present oneself inauthentically. In the workplace, underrepresented minorities are often seen as representatives of their group by others and expected to behave in ways that may feel inauthentic, to confirm others’ stereotypical beliefs about their group (Athanassiades, 1974; Kanter, 1977). Faced with this pressure, minorities do sometimes behave inauthentically to achieve relevant goals or comply with situational norms (Kirby, Rego, & Kaiser, 2020; Pickett et al., 2002; Sinclair & Huntsinger, 2006; Snyder et al., 1977); also see (Baumeister, 1982; Jones & Pittman, 1982; Schlenker, 1980), through strategies such as self-stereotyping, or applying perceivers’ cultural stereotypes to themselves (Hogg & Turner, 1987; Sinclair & Huntsinger, 2006).

These authenticity pressures may disproportionately impact those weakly identified with their group because they are especially likely to engage in identity management strategies to comply with contextual expectations (e.g., emphasizing or downplaying group identity; Barreto & Ellemers, 2000; Ellemers et al., 2002; Pickett et al., 2002; Spears et al., 1997). This is particularly true when their behavior is accountable (Ellemers et al., 1999), like in a workplace, and when neither group identity nor the individual self is under threat. Under these circumstances, they may strategically assert group identity or the individual self, depending on what is contextually appropriate. For example, when learning that their group is perceived positively, weakly identified members assert their group identity through increased self-stereotyping (Spears et al., 1997). Strongly identified group members, on the contrary, are relatively stable, behaving authentically and asserting their identity regardless of whether the group is perceived negatively or positively. Thus, multicultural and colorblind approaches may send messages about identity-based expectations for minorities, and weakly identified minorities may be most likely to alter their behavior to comply.

## Present Research

We present five experiments with online community samples of African Americans to examine how multicultural and colorblind approaches to organizational diversity shape our three key measures for strongly and weakly racially identified African Americans in a hiring context: prototypicality pressure, authenticity, and anxiety. We also examine their impact on two secondary measures exploring downstream implications: self-stereotyping and hiring desirability. Although some research has examined affective reactions to different diversity approaches (e.g., Plaut et al., 2009; Purdie-Vaughns et al., 2008), this is the first to examine fit between diversity approaches and individual characteristics and its impact on authenticity in particular.

### Hypothesis 1

A company that advocates managing diversity through multiculturalism will elicit greater prototypicality pressure relative to a company advocating colorblindness or no particular strategy (control condition), regardless of participants’ level of racial identification (Experiment 1). We expect similarities across the colorblind and control company contexts because colorblindness has historically been the default approach in American institutions (Plaut, 2002; Schofield, 1986; Shweder, 1991).

### Hypothesis 2

**Figure 1.**
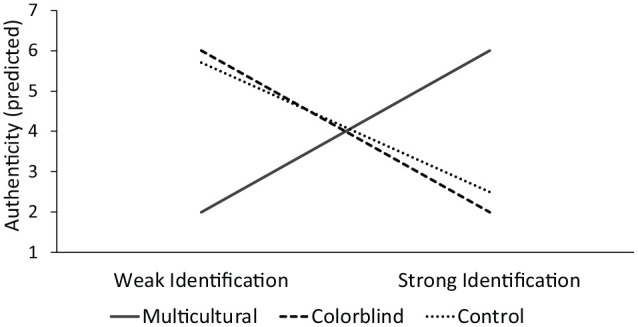
Predicted results for the authenticity dependent measure.

Among weakly racially identified minorities, a multicultural company will lead to greater anxiety and less authenticity relative to a colorblind or neutral control company (Experiments 1, 3, 4, and 5). Conversely, among strongly racially identified minorities, a multicultural company will lead to less anxiety and more authenticity relative to a colorblind or neutral control company (see Figure 1 for a visualization of predicted authenticity results).

### Hypothesis 3

**Figure 2.**
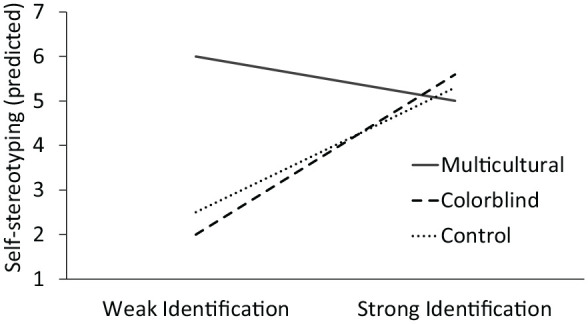
Predicted results for the self-stereotyping dependent measure.

We also examine potential consequences of reduced feelings of workplace authenticity. With respect to strategic self-stereotyping, we predict that among weakly racially identified minorities, a multicultural company will increase strategic self-stereotyping relative to a colorblind company or a control group because weakly identified minorities are more likely to engage in identity management strategies (Experiments 2–5). Because racial minorities tend to reduce self-stereotyping in the workplace by default (i.e., code switching; Debose, 1992), it is not clear whether they will further reduce self-stereotyping in the colorblind relative to control condition. We also predicted that strongly identified minorities would not adjust their levels of self-stereotyping in response to the diversity approaches because they tend to express their group identity regardless of strategic concerns (see Figure 2 for a visualization of predicted self-stereotyping results).

### Hypothesis 4

Finally, we predicted that reduced feelings of authenticity and increased anxiety would leak out in professional contexts, leading those participants to make worse impressions and experience worse hiring outcomes (Experiment 5). In other words, at a multicultural company, weakly racially identified minorities would be judged as less desirable applicants relative to those in the colorblind or control company. Conversely, those strongly identified would be judged as more desirable applicants at a multicultural relative to a colorblind or control company. Table 1 shows an overview of our predictions for all measures.

**Table 1.** Predictions for All Dependent Variables in the Multicultural Relative to Colorblind Conditions.

Dependent measure	Moderation by racial identification	Weakly identified	Strongly identified
Prototypicality pressure	0	↑	↑
Authenticity	**√**	↓	↑
Anxiety	**√**	↑	↓
Self-stereotyping	**√**	↑	0
Hiring desirability	**√**	↓	↑

*Note*. The moderation by racial identification column indicates whether moderation is predicted (with a √). The weakly and strongly identified columns indicate predictions for simple effects of diversity condition. ↑ indicates that we expect the multicultural condition to increase scores on the dependent measure relative to the colorblind condition. For example, we expect increased feelings of anxiety among weakly identified participants in the multicultural condition. ↓ indicates that we expect the multicultural condition to decrease scores on the dependent measure. 0 indicates that we expect no difference across conditions.

### Meta-Analytic Approach

To simplify the presentation and determine the overall, cumulative pattern of results, we conducted a meta-analysis across all five studies. Although it can be more difficult to study racial minority and other underrepresented groups while ensuring adequate statistical power (see Cortland et al., 2017; Fraley & Vazire, 2014, for a discussion of this issue), a meta-analytic approach can increase the confidence in findings. This is especially important when studying complex individual differences in minority populations, which are so critical to understanding the identity concerns experienced by minorities (e.g., Sellers & Shelton, 2003). Consistent with recommendations (Goh et al., 2016; Lakens & Etz, 2017), we reported all studies we conducted testing the research questions.

## Overview of Method

Because the study procedures were almost identical across experiments, we describe the method in full only for Experiment 1 (but see Supplemental Material for full details—we have disclosed all measures, manipulations, and exclusion criteria for all studies). For each subsequent experiment, we give a brief overview of its goals and describe any substantive changes that were made to the procedure (e.g., the addition of any key dependent variables). Table 2 contains a summary of this information, including sample characteristics and information about which dependent variables were included in each experiment.

**Table 2.** Sample Characteristics and Dependent Measure Overview Across Studies.

	Study					Meta-analysis
	1	2	3	4	5	
Sample characteristics						
Recruitment	PI	PI	MTurk	PI	PI	—
*N* African Americans	256	136	352	368	204	1,316
*N* Whites	—	1,487	—	—	—	—
% Female	69%	74%	68%	67%	72%	—
Stopping rule	291 (power analysis)	70 per condition	As many as opt to participate in Wave 2	375 (power analysis)	70 per condition	—
Dependent variable						
Prototypicality pressure	**•**					
Authenticity	**•**		**•**	**•**		**•**
Anxiety	**•**		**•**	**•**		**•**
Essay authenticity/anxiety					**•**	**•**
Hiring desirability					**•**	
Trait self-stereotyping		**•**	**•**		**•**	**•**
Activity self-stereotyping		**•**	**•**	**•**		**•**
Interest in Black Network			**•**	**•**		**•**

*Note*. The sample characteristics section gives information about how participants were recruited (PI = Project Implicit), the sample size for each group, and how we determined the sample size for each study (stopping rules). Stopping rules were determined before data analysis with the exception of Study 2, where we conducted a preliminary analysis and then collected approximately 40 additional participants to increase statistical power. In some cases, our final sample was lower than our stopping rule because of unanticipated exclusions (participants not meeting the prescreening criteria specified). The final section includes information about which dependent variables were included in each experiment.

### Experiment 1

In the first experiment, we examined how multicultural and colorblind approaches to organizational diversity shape prototypicality pressure, authenticity, anxiety, and how that might differ among strongly and weakly racially identified minorities. African Americans imagined interviewing at a company that advocated managing diversity either through multiculturalism or colorblindness or one that gave no information about the diversity approach (control condition) and then responded about prototypicality pressure in that context, as well as anticipated anxiety and authenticity. We predicted that weakly and strongly identified minorities would be equally likely to perceive multiculturalism (compared with colorblindness) as producing prototypicality pressure but that weakly identified minorities would be more likely to have a negative reaction to these concerns, feeling increased anxiety and inauthenticity in a multicultural context. We expected the reverse for those strongly identified with their group that they would have a positive reaction to multiculturalism relative to colorblindness, consistent with past findings (Purdie-Vaughns et al., 2008).

#### Participants

A total of 408 African American visitors to the Project Implicit website (https://implicit.harvard.edu), who volunteered to participate in implicit social cognition research, were randomly assigned to complete the present study from a pool of available studies. Five were excluded because 10% or more of their Implicit Association Test (IAT) trials were faster than 300 ms. Of the remaining 403 participants, 256 reached the end and completed the main independent and dependent measures (177 women, 79 men; *M*_age_ = 34.49, *SD* = 13.75; 95% had completed some college or a higher level of education). This is consistent with Project Implicit completion rates that typically range from 50% to 70%. To maximize statistical power, we retained partial data for those not fully completing the study, resulting in varying degrees of freedom in analyses (as in subsequent studies as well). Accounting for attrition, sensitivity analyses showed that we had adequate power (π = .80) to detect a slope difference by condition (i.e., an interaction between racial identification and condition) of β = 0.38 for authenticity. Full details of all sensitivity analyses are included in Supplemental Table S1.

#### Procedure

Participants first read a recruitment brochure from a consulting company called CCG Business Consulting (modeled after brochures from Purdie-Vaughns et al., 2008, but adhering to the operationalizations used by Plaut et al., 2011; see Supplemental Material) and were instructed to consider working for CCG. The diversity approaches were manipulated via the content of the brochure. In the colorblind condition, the brochure emphasized that the company’s ethnically diverse workforce should embrace their similarities and that their race, ethnicity, and culture are immaterial. In the multicultural condition, the brochure instead encouraged participants to embrace their differences and emphasized that their race, ethnicity, and culture are an asset. We modified the original manipulations from Purdie-Vaughns and colleagues (2008) to make the colorblind and multicultural statements as parallel as possible^4^—only 7% of the words differed across the multicultural and colorblind conditions. In the control brochure, no information was given about the company’s diversity approach, but all other information about CCG was identical.

Participants next imagined that they were interviewing at CCG and responded to six items assessing prototypicality pressure (adapted from Sekaquaptewa et al., 2007; for example, “CCG would be more likely to hire me if I conformed to their expectations about my racial/ethnic group”; α = .82; see Supplemental Material for full scale items), four items on state authenticity developed specifically for this study (e.g., “I would be my true self at the CCG interview”; α = .84), and three items about state anxiety in the interview scenario developed specifically for this study (e.g., “I would feel anxious at the CCG interview”; α = .77; see Supplemental Material for results of the factor analysis). Scale endpoints were 1 (*strongly disagree*) to 7 (*strongly agree*), and the measures were scored so that higher values indicated greater anxiety, greater authenticity, and greater prototypicality pressure.^5^ They also completed an IAT because Project Implicit volunteers visit the website to learn about their implicit attitudes. Because the measure was not central to hypotheses in Experiment 1, and for the sake of brevity, it is only discussed in the Supplemental Material—there was no effect of diversity condition for this measure in any studies.

Next, to examine whether participants’ level of racial identification would moderate how they responded to diversity condition, they completed the four items of the centrality subscale of the collective self-esteem scale (Luhtanen & Crocker, 1992) on a 1 (*strongly disagree*) to 7 (*strongly agree*) scale (α = .77). Although some research has clustered together several identification dimensions (see Leach et al., 2008, for a discussion) rather than focusing on the particular subcomponent of interest (Kaiser & Spalding, 2015; Operario & Fiske, 2001; Sellers et al., 1997), we focused exclusively on centrality. This was important for two reasons: (a) to ensure that our moderator variable was distinct from one of our primary dependent measures, self-stereotyping, which is sometimes considered a component of identification (Leach et al., 2008; see Derks et al., 2011, 2015; Spears et al., 1997), and (b) unlike other components of identification, the centrality dimension is theorized to be stable across situations (Major et al., 2002; Sellers et al., 1998), so is particularly appropriate as an individual difference measure.

We measured identification at the end of the study and after the manipulation to facilitate our cover story around the hiring scenario and avoid making our hypotheses transparent. In line with theorizing (Sellers et al., 1998), diversity condition did not affect participants’ level of racial identification in this study, *F*(2,256) = 0.19, *p* = .830, or any other studies.

Finally, to determine whether participants interpreted the manipulation as intended, they responded to the following item: “To what extent does CCG focus on the differences between different racial and ethnic groups?” (1 = *focuses not at all*, 2 = *focuses slightly*, 3 = *focuses moderately*, 4 = *focuses a great deal)*.

### Experiment 2

In Study 2, we examined the potential consequences of reduced feelings of workplace authenticity. Specifically, we expected that weakly identified African Americans’ concerns about being authentic in multicultural organizations may reflect perceived pressure to behave in line with stereotypes of their group. Thus, Experiment 2 examined whether diversity approaches would lead participants, particularly those who are weakly identified, to adjust their self-stereotyping in workplace contexts (Hypothesis 3). Specifically, we predicted that among weakly racially identified minorities, a multicultural company would increase strategic self-stereotyping relative to a colorblind company or a control group because weakly identified minorities are more likely to engage in identity management strategies.

Explained another way, under neutral circumstances, the stronger a person identifies with their group, the more likely they are to assert that identity through strategies such as self-stereotyping; however, this pattern can change when impression management concerns are salient. Because those who are weakly identified are particularly likely to respond to impression management concerns (Ellemers et al., 2002; Pickett et al., 2002; Spears et al., 1997), they might self-stereotype at similar levels as those strongly identified in the multicultural relative to colorblind context.

We also included a White American sample in this study to examine whether diversity approaches create identity-related pressure exclusively for racial minorities. Because Whites typically feel excluded from diversity structures (Dover et al., 2016; Plaut et al., 2011), we did not expect that they would interpret multicultural and colorblind approaches as a model for how they should behave. These results are only presented in the Supplemental Material.

We did not measure authenticity, anxiety, or prototypicality pressure in this experiment to keep the study length manageable for our volunteer participants. We also did not include a control condition in this study. Aspects of the methodology not described in the previous experiment are described below.

#### Participants

A total of 1,487 White and 136 African American visitors to the Project Implicit website participated in this experiment. Further details about participants and results for White participants are described in the Supplemental Material.

### Additional Measures

#### Activity self-stereotyping

After reading the CCG brochure, participants imagined that they were interviewing at CCG and had been asked to complete a set of questionnaires for the organization. The questions ostensibly assessed their personality and interests but were actually measuring self-stereotyping. They first responded to questions such as “How much do you enjoy the following activities?” on a 1 (*not at all*) to 7 (*extremely*) scale about 35 activities and interests (Steele & Aronson, 1995), nine of which were considered stereotypical of African Americans (α = .75; rap/hip-hop, football, sports, basketball, talking, gospel music, physical education, athletics, track; see Supplemental Material for information about pilot testing of items) and 26 of which served as filler items.

#### Trait self-stereotyping

A second self-stereotyping measure was embedded with stereotypically African American traits used in previous research (Judd et al., 1995; Wolsko et al., 2000), as well as filler items. Participants responded about the extent to which 32 positive and negative traits described them, of which five were positive stereotypes of African Americans (streetwise, humorous, athletic, musical, emotionally expressive; α = .56; see Supplemental Material for information about pilot testing of items). Scale endpoints were 1 (*not at all descriptive of me*) to 7 (*very descriptive of me*).

### Experiment 3

In an effort to replicate the findings from the first two experiments and to demonstrate these effects within a single study, Experiment 3 used a new sample to test effects on anxiety, authenticity, and self-stereotyping. We added a supplement to our previous measures of self-stereotyping, which is described below.

#### Participants

We recruited 352 African American Amazon Mechanical Turk workers through Turkprime, an online crowdsourcing platform that allows for recruitment of participants with specified demographic criteria (see Litman et al., 2017, for more information), in exchange for US$2.05. Further details are included in the Supplemental Material.

### Additional Measures

#### Interest in Black CCG Network

As a supplement to our previous measures of self-stereotyping, participants responded to “How interested would you be in the following CCG organizations?” on a 1 (*not at all interested*) to 5 (*extremely interested*) scale. Although we were only interested in their response to “the Black CCG Network,” potential organizations included Non-Profit Consulting Society, Women at CCG, Future Leaders Society, Asian Consulting Society, Black CCG Network, and Latino/a Mentoring Group (in that order).

### Experiment 4

Due to unexpected findings in Experiment 3 with a sample from Mechanical Turk, we returned to a Project Implicit Sample in Experiment 4 to examine whether we would replicate the effects of Experiments 1 and 2 with participants recruited the same way as the original samples. The methodology was otherwise identical to Experiment 3 and included 368 African American participants. Further details are included in the Supplemental Material.

### Experiment 5

Experiment 5 examined another potential consequence of the mismatch between multicultural contexts and those weakly identified with their racial group (and colorblind contexts and those strongly identified): negative hiring outcomes. Participants again considered employment at a multicultural relative to a colorblind or control company, but instead wrote an open-ended essay describing themselves. We then coded essays for evidence of authenticity and anxiety and asked an independent sample of participant raters to read the essays and indicate their willingness to hire each applicant. We describe any new methodological information below.

#### Participants

A total of 204 African American visitors to the Project Implicit website participated in this experiment. Further details are included in the Supplemental Material.

#### Procedure

After reading a CCG recruitment brochure, participants were told that CCG would like to know more about them and that their responses could inform the types of events organized by Human Resources in the future. They responded to an essay prompt about their favorite activities and interests, as well as their personality characteristics, which we later coded for authenticity and anxiety.

#### Authenticity essay coding

Four research assistants (one African American, one Asian American, one White, one White/Asian biracial) coded the essays for authenticity on a 1 (*not at all*) to 5 (*extremely*) scale: “This person is being authentic” and “This person is being genuine.” Coder responses had moderate to good interrater reliability (intraclass correlation coefficient [ICC] = .75; Koo & Li, 2016) and were averaged to create a measure of authenticity for each essay. Coders were blind to all hypotheses and the experimental condition of the essay writer, but, because racial identity is generally visible when forming impressions of others, we informed them that all essays were written by African American participants.

#### Anxiety essay coding

Two research assistants (one Asian American, one White) coded the essays for anxiety on a 1 (*not at all*) to 5 (*extremely*) scale: “This person seems anxious”; “This person seems nervous”; and “This person seems comfortable” (reverse coded). Coder responses for each essay had poor to moderate interrater reliability (ICC = .46; see Koo & Li, 2016, for guidelines) and were averaged to create a measure of anxiety for each essay. Coders were blind to all hypotheses and the experimental condition of the essay writer, but, because racial identity is generally visible when forming impressions of others, we informed them that all essays were written by African American participants.

#### Hiring outcome ratings

We recruited a separate sample of 125 University of Washington undergraduate students (54 White, 32 Asian, 20 Multiracial, six Latino, four Black, two American Indian, two Other, and five unspecified; 72 females, 50 males, three unspecified) to read the essays and rate the extent to which they saw the essay writer as a desirable job applicant. Participants received extra course credit in their psychology courses in exchange for participation. The raters were told to imagine that they were a hiring manager and to read several short paragraphs in which people described themselves. Because racial identity is generally visible in hiring interviews, we made this information available to raters to increase ecological validity. They learned that the essays were divided into demographic subgroups and the candidates they had been assigned to evaluate were African Americans between the age of 18 and 40 of any gender.

Each rater was randomly assigned to read a subset of 40 different essays, and they were blind to all hypotheses and condition of the essay writer. Randomization was constrained so that each essay would be rated an approximately equal number of times. Due to missing data, each essay was rated between 6 and 10 times (*M* = 8.89). Raters responded to the following questions on a 1 (*not at all*) to 7 (*extremely*) scale: “How likely would you be to invite this person for an interview?; How likely would you be to hire this person?” and “How well would this person fit at the company?”

The rater responses for each essay were averaged to create a measure of hiring desirability (α = .99) for each essay. We could not run interrater reliabilities because all 125 participants rated a different subset of essays (in line with Kaiser & Miller, 2001), resulting in substantial missing data for every rater. However, the relatively large number of raters for each essay (approximately nine) helps promote reliability and more certainty in the overall impressions reported.

## Results

We present meta-analyzed results when possible, but some key dependent measures could not be meta-analyzed because they were only measured once: prototypicality pressure and hiring desirability. In these cases, we present the original moderated regression analyses. After discussing manipulation checks and our analytic strategy, we first present analyses for (a) key measures analyzed individually (prototypicality pressure), (b) key measures aggregated meta-analytically (authenticity and anxiety), and (c) secondary measures that explored downstream implications of the key findings: self-stereotyping (aggregated meta-analytically) and hiring desirability (analyzed individually). All individual study results are fully described in tables, figures, and in the Supplemental Material, and all data sets are available at https://osf.io/8nh2b/?view_only=b058e48b9ecc4fb791c148d7c118f8f2.

### Manipulation Checks

Across all studies, participants perceived a significantly greater focus on group differences in the multicultural than in the colorblind condition, *p*s < .001, and in the control condition, *p*s ≤ .001, when it was included (Studies 1, 3, 4, and 5). However, participants did not perceive a difference in how much the control and colorblind companies focused on group differences, *p*s > .092.

### Analytic Strategy

#### Moderated regression

To test the main hypotheses for individual studies, two dummy coded variables for diversity condition were entered into the first step of a hierarchical linear regression model in which multiculturalism, the reference group, was always coded as 0. Thus, one variable compared the multiculturalism condition with the control condition (coded as 1), if included in the study, and the other variable compared the multiculturalism with the colorblind condition (coded as 1). Centered racial identification scores were also entered into the first step of the model. All two-way interactions were entered into the second step. If the *R*-squared change (Δ*R*^2^) corresponded to *p* < .05 for a step of the regression model, we followed up with simple effects analyses for the highest-order significant interactions using the PROCESS macro (Hayes, 2013). Thus, for any interactions that were significant (*p* < .05), we first broke down interactions for the multicultural relative to colorblind comparison. Next, we broke down interactions for the multicultural relative to control comparison. We also conducted a parallel regression analysis with the control condition as the reference group (to test the comparison between the colorblind and control group). For the sake of simplicity, we only discuss this comparison when it is statistically significant.

To conduct simple effects analyses, we used the Johnson-Neyman technique (Preacher et al., 2006). Rather than setting predefined values to represent “strong” and “weak” levels of racial identification (Aiken & West, 1991), the Johnson-Neyman technique determines the values of the moderator (racial identification) at which a significant difference across conditions emerges (*p* = .05), if at all. A benefit of this approach is that it provides a more complete description of the divergence of the slopes (i.e., simple effects), both above and below mean levels of the moderator variable, rather than restricting the description to probing at single data points (e.g., ±1 *SD* of the mean). We also conducted the simple effects analyses described below as part of the internal meta-analysis.

#### Meta-analytic strategy

In the internal meta-analysis, we examined state anxiety, authenticity, and self-stereotyping. We first ran a meta-analysis that compared the aggregate slopes (Pearson’s *r* converted to Fisher’s *Zr*; see Lipsey & Wilson, 2001) for the multicultural, colorblind, and control conditions to determine whether there was an overall interaction effect on anxiety, authenticity, and self-stereotyping. Next, we conducted simple effects meta-analyses separately for those weakly and strongly identified with their racial group for the dependent variables showing a significant interaction.

To calculate effect sizes for the simple effects meta-analyses, we defined “strong” and “weak” racial identification, respectively, as those who moderately agreed (6 on a 7-point scale) and moderately disagreed (2 on a 7-point scale) that their racial identity was important to them. Although it is customary to define these values as ±1 standard deviation of the moderator mean, this would have led to slightly different definitions of “strong” and “weak” identification across the studies. We instead chose static values that were a reasonable conceptual reflection of weak and strong identification, as recommended by Aiken and West (1991). Importantly, participant data points extended even beyond these chosen values when examining predicted slopes.

We converted the unstandardized regression coefficients from these simple effects to Cohen’s *d* using Lipsey and Wilson’s (2001) meta-analysis effect size calculator, which also requires the standard deviation of the dependent measure and the *n* for each condition as an input.^6^ When there were multiple measures for an experiment (e.g., both a trait and an activity self-stereotyping measure), we combined those into one effect size, in line with recommendations by Borenstein and colleagues (2009), to ensure that each effect size represented an independent sample. All meta-analyses were conducted with the MeanES.sps and MetaF.sps SPSS macros (Wilson, 2005) using method of moments. Because the methods were identical across studies and we only sought to describe the effect size of the present studies (see Goh et al., 2016), we used fixed effects models.

### Does Multiculturalism Create Prototypicality Pressure?

In Experiment 1, we anticipated that participants would perceive more race prototypicality pressure in the multicultural company context compared with colorblind and control companies, with no moderation by racial identification. This would show that participants interpret the approaches in similar ways. In a hierarchical moderated regression analysis, a main effect emerged between the multiculturalism condition relative to colorblindness and control. Specifically, participants perceived more prototypicality pressure in the multicultural company context (*M* = 3.93) compared with colorblind (*M* = 3.13) and control companies (*M* = 3.13). These effects were not moderated by racial identification (see Table 3), which suggests that the multicultural approach to diversity management heightened prototypicality pressure among participants irrespective of racial identification.

**Table 3.** Hierarchical Regression on Measures Analyzed Individually.

Predictor	Prototypicality pressure (Experiment 1)		Hiring desirability (Experiment 5)		Positive self-presentation (Experiment 5)	
	β	*p*	β	*p*	β	*p*
Step 1	Δ*R*^2^ = .10, *p* < .001		Δ*R*^2^ = .06, *p* = .011		Δ*R*^2^ = .01, *p* = .537	
Racial identification	−0.05	.453	0.25	.001	0.09	.245
Control (vs. multicultural)	−0.29	<.001	0.04	.603	0.03	.747
Colorblind (vs. multicultural)	−0.31	<.001	0.04	.628	−0.04	.689
Step 2	Δ*R*^2^ = .001, *p* = .844		Δ*R*^2^ = .05, *p* = .015		Δ*R*^2^ = .01, *p* = .391	
Control (vs. Multicultural) × identification	0.04	.573	−0.27	.009	−0.11	.321
Colorblind (vs. Multicultural) × identification	0.01	.907	−0.26	.016	−0.15	.188

*Note*. Regression coefficients are reported from the step on which each variable was first entered. The multicultural condition, the reference group in the regression, is always coded as 0, with control and colorblindness coded as 1.

### How Do Multiculturalism and Colorblindness Impact Anxiety and Authenticity?

We next examined the anxiety and authenticity dependent measures in an internal meta-analysis. We predicted that for weakly racially identified minorities, a multicultural company would lead to greater anxiety and less authenticity relative to a colorblind and control company. Conversely, we anticipated that strongly racially identified minorities would feel less anxious and more authentic at a multicultural company relative to a colorblind and control company. As shown in Table 4, our interaction hypotheses were confirmed for anxiety and authenticity in three of the four studies (Studies 1, 4, and 5, but not Study 3).

**Table 4.** Individual Study Results for Dependent Measures Comprising Meta-Analysis

		Authenticity		Anxiety		Activity Self-Stereotyping		Trait Self-Stereotyping	
Study	Effect	Low ID	High ID	Low ID	High ID	Low ID	High ID	Low ID	High ID
1	Diversity x Id β (*p)*	**−0.26 (.003)**		**0.23 (.007)**		—		—	
	Simple Effect *d* (*p*)	**0.84 (.008)**	**−0.37 (.069)**	**−0.81 (.001)**	0.27 (.182)	—	—	—	—
	J-N cutpoint	**3.46 (24%)**	**6.19 (14%)**	**3.61 (28%)**	—	—	—	—	—
2	Diversity x Id β (*p)*	—		—		**0.27(.021)**		**0.25 (.035)**	
	Simple Effect *d* (*p*)	—	—	—	—	**−0.65 (.031)**	0.11 (.604)	**−0.59 (.048)**	0.13 (.532)
	J-N cutpoint	—	—	—	—	**3.51 (27%)**	—	**2.44 (10%)**	—
3	Diversity x Id β (*p)*	.04 (.613)		.12 (.112)		−.09 (.249)		−.07 (.387)	
	Simple Effect *d* (*p*)	**−0.57 (.049)**	**−0.38 (.018)**	−0.13 (.641)	**0.40 (.014)**	0.17 (.555)	−0.23 (.160)	0.02 (.937)	−0.28 (.092)
	J-N cutpoint	—	—	—	—	—	—	—	—
4	Diversity x Id β (*p)*	**−0.18 (.022)**		**0.23 (.002)**		−0.05 (.529)		—	
	Simple Effect *d* (*p*)	**0.61 (.022)**	−0.20 (.214)	**−0.84 (.003)**	**0.21 (.177)**	0.41 (.114)	0.20 (.201)	—	—
	J-N cutpoint	**3.24 (15%)**	—	**4.04 (35%)**	**6.69 (82%)**	—	—	—	—
5	Diversity x Id β (*p)*	**−0.24 (.033)**		0.21 (.061)		—		**0.30 (.003)**	
	Simple Effect *d* (*p*)	0.50 (.163)	**−0.64 (.030)**	−0.61 (.10)	0.33 (.245)	—	—	**−1.11 (.002)**	**0.41 (.110)**
	J-N cutpoint	—	**5.43 (31%)**	—	—	—	—	**3.81 (37%)**	**6.55 (6%)**

*Note*. The studies are presented in this order for narrative purposes, but the original order of studies was 2, 5, 1, 3, and 4. Study 2 results are reported when excluding White participants in order to make the effect sizes comparable for meta-analytic purposes. Diversity corresponds to the comparison of the multicultural (0) relative to the colorblind (1) diversity conditions, but statistics for the control comparison are presented in the meta-analysis and online supplement. Simple effects compare the multicultural to colorblind conditions when probed at racial identification values of 2 (moderately disagree) and 6 (moderately agree), to be consistent across experiments. The J-N (Johnson-Neyman) cutpoint designates the value of racial identification at which the effect of diversity condition became statistically different at *p* < .05 and what percentage of participants fell below (for low identification) or above (for high identification) that value. If no value is listed, there was no point at which the difference between conditions became statistically significant within the observed range of racial identification or the interaction was not significant. The simple effects statistics presented are from analyses that did not include the control groups, as this was necessary in order to run the Johnson-Neyman technique in PROCESS and it also ensured comparable coefficients for aggregation in the meta-analysis. Statistically significant (*p* < .05) effects are in bold, including simple effects that were significant using the J-N technique, even if not significant at “low” and “high” identification.

**Figure 3.**
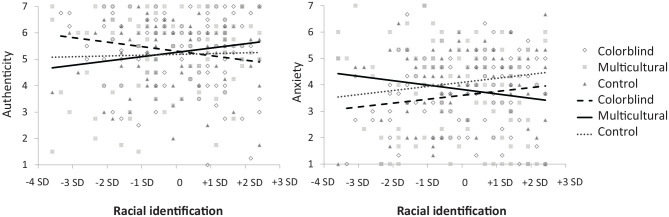
Authenticity and anxiety among African American participants varying in racial identification in Experiment 1.

The meta-analytic results bore out these predictions as well, showing that the relationship between racial identification and authenticity, *Q*(2)_*B*_ = 13.90, *p* = .001, and anxiety, *Q*(2)_*B*_ = 21.00, *p* < .001, differed by condition (see Table 5 for pairwise breakdowns). The simple effects confirmed predictions (see Tables 6 and 7), with the exception of the pattern of the control condition contrasts. Specifically, strongly and weakly identified African Americans had opposing reactions to diversity approaches, with those weakly identified feeling more authenticity and less anxiety in a colorblind context relative to control and multicultural context. However, those strongly identified felt more authenticity and less anxiety in a multicultural relative to control and colorblind context. Figure 3 shows the typical pattern of the authenticity and anxiety results, using the data from Experiment 1.

**Table 5.** Meta-Analysis of Racial Identification Slope Differences by Diversity Condition (Interaction Effects).

Test	Measure	*Q*(1)_*B*_	*p*
Colorblind (1) vs. multicultural (0)	Authenticity	12.39	<.001
	Anxiety	20.93	<.001
	Self-stereotyping	1.61	.205
Colorblind (1) vs. control (0)	Authenticity	8.00	.005
	Anxiety	6.38	.012
	Self-stereotyping	0.30	.581
Control (1) vs. multicultural (0)	Authenticity	0.34	.557
	Anxiety	3.74	.053
	Self-stereotyping	0.40	.526

*Note.* These analyses examine whether the relationship between racial identification and the dependent variables (Pearson’s *r*) differed in the multicultural, color- blind, and control conditions, to determine whether there was an overall interaction effect.

**Table 6.** Meta-Analysis of the Relationship Between Racial Identification and Dependent Measures by Diversity Condition (Simple Slope Analysis).

Test	Measure	Mean *r* (simple slope)	95% CI	*p*
Multicultural	Authenticity	.16	[.06, .25]	.002
	Anxiety	−.17	[−.26, −.07]	<.001
	Self-stereotyping	.04	[−.06, .13]	.472
Colorblind	Authenticity	−.10	[−.20, .004]	.060
	Anxiety	.16	[.06, .26]	.002
	Self-stereotyping	.13	[.02, .23]	.015
Control	Authenticity	.11	[.01, .22]	.034
	Anxiety	−.03	[−.13, .08]	.611
	Self-stereotyping	.09	[−.03, .20]	.148

*Note.* CI = confidence interval.

**Table 7.** Meta-Analysis of the Effects of Diversity Condition for African Americans Low and High in Racial Identification (Simple Effects Analysis).

Comparison	Measure	Experiments contributing data	Total *n*	Mean *d*	95% CI	*p*
Weakly identified African Americans						
Colorblind (1) vs. multicultural (0)	Authenticity	1,3,4,5	791	0.30	[0.15, 0.44]	<.001
	Anxiety	1,3,4,5	791	−0.58	[−0.72, −0.44]	<.001
Colorblind (1) vs. control (0)	Authenticity	1,3,4,5	742	0.45	[0.31, 0.60]	<.001
	Anxiety	1,3,4,5	742	−0.50	[−0.65, −0.35]	<.001
Control (1) vs. multicultural (0)	Authenticity	1,3,4,5	769	−0.18^a^	[−0.32, −0.04]	.014
	Anxiety	1,3,4,5	769	−0.09	[−0.23, 0.06]	.236
Strongly identified African Americans						
Colorblind (1) vs. multicultural (0)	Authenticity	1,3,4,5	791	−0.36	[−0.50, −0.22]	<.001
	Anxiety	1,3,4,5	791	0.30	[0.16, 0.44]	<.001
Colorblind (1) vs. control (0)	Authenticity	1,3,4,5	742	−0.05	[−0.20, 0.09]	.466
	Anxiety	1,3,4,5	742	0.01	[−0.14, 0.15]	.945
Control (1) vs. multicultural (0)	Authenticity	1,3,4,5	769	−0.30	[−0.44, −0.16]	<.001
	Anxiety	1,3,4,5	769	0.30	[0.16, 0.45]	<.001

*Note*. The direction of effect sizes reflects the original coding in the studies. For example, multiculturalism was coded as 0 and colorblindness as 1 in the primary analyses, so a negative effect size for that comparison on authenticity reflects increased authenticity in the multicultural relative to colorblind condition. CI = confidence interval.

a Indicates that the direction of the effect is opposite of hypotheses.

Taking all results together so far, participants in the multicultural condition detected more pressure to be prototypical group members relative to those in the colorblind and control condition, regardless of levels of racial identification. However, only weakly identified African Americans responded to this pressure, showing more comfort in the colorblind than multicultural and control contexts. Strongly identified participants were instead unaffected by prototypicality pressure, experiencing more comfort in the multicultural relative to colorblind and control conditions.

### What Are the Downstream Implications of Increased Authenticity and Anxiety?

#### Self-stereotyping

We next examined the self-stereotyping measures meta-analytically, as one potential outcome of increased authenticity and anxiety. We predicted that among weakly (but not strongly) racially identified minorities, a multicultural company would increase strategic self-stereotyping relative to a colorblind company because weakly identified minorities are susceptible to engaging in identity management strategies. As shown in Table 3, our hypotheses were only confirmed for self-stereotyping in two of the four studies (Studies 2 and 5, but not Studies 3 and 4), and the predictions did not ultimately bear out in the internal meta-analysis (see Tables 5–7). Specifically, the relationship between racial identification and self-stereotyping did not significantly differ by condition, *Q*(2)_*B*_ = 1.61, *p* = .446, so we did not further probe the simple effects.

#### Hiring desirability (Experiment 5)

**Figure 4.**
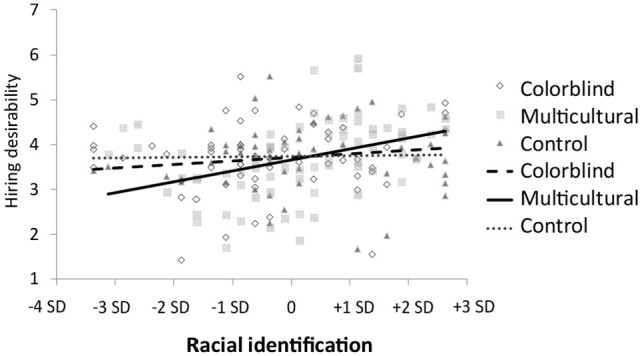
Hiring desirability of African American participants varying in racial identification in Experiment 5. Independent raters judged participants’ essay responses.

Experiment 5 examined another potential consequence of lack of fit between diversity approaches and racial identification: negative hiring outcomes. We expected that in the multicultural condition, weakly identified participants would be seen as less desirable applicants than those in the colorblind and control conditions. This would happen because their anxiety and inauthenticity would leak out in their self-descriptions. Our regression analysis showed that this hypothesis was confirmed for both the multicultural relative to colorblind, *b* = 0.66, *SE* = 0.27, *p* = .015, and control comparisons, *b* = 0.78, *SE* = 0.30, *t*(183) = 2.26, *p* = .010 (see Figure 4 and Table 4 for overall interaction statistics).^7^ The interaction for the colorblind versus control comparison was not statistically significant, β = 0.03, *t*(169) = 0.29, *p* = .771.

For strongly identified participants in the multicultural condition, we hypothesized that they would instead be judged as more desirable applicants than those in the colorblind and control conditions. Although this was again the observed pattern, this difference did not reach conventional levels of statistical significance, *p*s > .146. Overall, this pattern of results shows that weakly identified participants experience worse hiring outcomes in the multicultural relative to colorblind and control condition, which somewhat matches the findings for authenticity and anxiety. However, the findings should be interpreted cautiously because hiring outcomes were only examined in a single study.

## General Discussion

Diversity initiatives have proliferated in recent years. The multicultural approach, which values and encourages the expression of group differences, has received considerable attention because it offers significant benefits to racial minorities relative to colorblindness. However, the present research tested the hypothesis that some of its benefits (and that of colorblindness) would depend on minorities’ levels of racial identification. We tested this hypothesis with a large sample (*N* = 1,316) of African American participants, who are underrepresented in the psychology literature and in many workplaces (U.S. Census Bureau, 2012). Indeed, multiculturalism increased prototypicality pressure in one study (relative to colorblind and control company contexts), irrespective of participants’ racial identification (Experiment 1), but participant reactions to this pressure did depend on racial identification (Experiments 1–3). Whereas strongly identified minorities felt more authentic and less anxious in the multicultural relative to the colorblind and control conditions, weakly identified minorities felt more authentic and less anxious in the *colorblind* relative to multicultural and control conditions in an internal meta-analysis. Thus, feelings of authenticity when considering an organizational context depend on fit between racial identification and the diversity approach, consistent with theorizing about goal fit as a route to authenticity (Schmader & Sedikides, 2018).

Although our hypotheses focused on multiculturalism as causing differential reactions among weakly and strongly identified minorities, comparisons with the control condition told a slightly different story. Rather than weakly identified minorities feeling uncomfortable with multiculturalism, they received an authenticity boost from colorblindness. Similarly, rather than strongly identified minorities feeling uncomfortable with colorblindness, they received an authenticity boost from multiculturalism (but see Purdie-Vaughns et al., 2008, which suggests this may depend on representation).

Furthermore, these feelings of authenticity and anxiety were apparent in professional self-descriptions, tentatively leading to less hiring desirability among weakly identified participants in a multicultural context. This latter finding was only examined in a single study (as was prototypicality pressure) and only partially matched the pattern of differences for authenticity and anxiety. Thus, it should be interpreted cautiously unless replicated in future research.

### Self-Stereotyping

We also hypothesized that multicultural approaches would promote self-stereotyping, but only among those weakly identified with their racial group. Specifically, strategic self-stereotyping and stereotype distancing would only occur among weakly racially identified minorities because, compared with strongly identified minorities, they are more likely to engage in identity-related impression management strategies to obtain desired outcomes (Ellemers et al., 2002). Although there was evidence supporting this hypothesis in Experiments 2 and 5, the effects did not hold in Experiment 3 or 4, and the overall meta-analytic effect was not statistically significant in either direction.

### Caveats and Limitations

We used the same multicultural and colorblind manipulations across all experiments. Although this facilitated direct replication, it is important to understand whether these results generalize to different ways of framing multiculturalism and colorblindness, particularly ones that focus less narrowly on racial and ethnic differences (Purdie-Vaughns et al., 2008).

Despite adapting the manipulations, supplemental measures included in the study and reported in the Supplemental Material showed evidence for replication of previous effects in the literature. For example, participants believed that they would be stereotyped less and had fewer concerns about being a good representative of their group at the multicultural relative to colorblind or control company, which is consistent with the finding that African Americans trust multicultural organizations more (Purdie-Vaughns et al., 2008). These findings are especially interesting given the (seemingly conflicting) findings for prototypicality pressure, showing that multiculturalism increases prototypicality pressure relative to colorblindness. This inconsistency may reflect the neutral phrasing of prototypicality pressure. In other words, participants may believe that they will be treated more fairly at a multicultural company, while also acknowledging that it will be more desirable to present themselves prototypically at that same organization. However, for weakly identified participants, the perception that they will be treated fairly does not translate into feelings of state authenticity and reduced anxiety at the organization, which is consistent with arguments that authenticity is distinct from constructs like belonging and anticipated organizational treatment (Schmader & Sedikides, 2018).

A limitation of the present research is that all participants—with the exception of those in Experiment 3—were from the Project Implicit website, which attracts relatively highly educated participants. Future research should examine how diversity approaches affect a more nationally representative sample of racial minorities, as well as minority groups other than African Americans.

Nonetheless, Project Implicit sampling has benefits. The site has more representative samples than the university student samples typically used in social psychology research. In addition, Project Implicit may have tapped into a wider range of racial identification levels than do other recruitment tools. Our Project Implicit samples reported a mean racial identification of 4.58 on a 1 to 7 agreement scale (4.44–4.87; *SD* = 1.50), whereas the Mechanical Turk sample in Experiment 3 had a mean racial identification of 4.84. Similarly, Shelton and Sellers (2000), who examined racial identification stability in a sample of undergraduate African Americans, reported a higher mean racial identification ranging from 4.9 to 5.1 (*SD* = 0.9), using a similar 1 to 7 agreement scale as ours. Given that the present samples may be somewhat less identified with their racial group and show more dispersion than some university student samples, the psychological research literature may be neglecting individuals who are weakly identified with their racial or ethnic group. This is one potential explanation for the fact that the pattern of moderation observed in most of the present studies did not replicate in the Mechanical Turk sample.

A final limitation is that the primary effects of interest on authenticity and anxiety accounted for a relatively small proportion of the variance explained (2%–5%). However, seemingly small effect sizes can have a meaningful impact in the real world (Rosenthal & Rubin, 1982). In a simulation study, gender bias that initially accounted for only 1% of the variance in performance scores led to substantial promotion inequality, where women comprised only 35% of top-level positions (Martell et al., 1996).

### Implications and Future Directions

These findings could have important downstream consequences for some minority groups. People desire to be seen in ways that are consistent with their own self-views (Swann & Read, 1981) and have lowered self-esteem and positive affect when they behave inauthentically (Harter, 2002). Minorities also experience negative affect in intergroup interactions when they behave inauthentically (Newheiser & Barreto, 2014). Thus, inauthentic behavior or discomfort in workplace contexts may lead to both negative psychological experiences and harm employee relations. Furthermore, workplace stress and anxiety can lead to lowered efficiency, job satisfaction and retention, performance, and well-being (Cavanaugh et al., 2000; Linden & Muschalla, 2007).

The present research has important implications for organizational and institutional best practices. Although we have demonstrated that multiculturalism does not have the same benefits for weakly identified minorities as it does for those strongly identified, it also did not have clear negative implications and could potentially be adjusted to be more beneficial. One possibility is to combine multiculturalism with other strategies that acknowledge the individual self. *Identity safety* is a diversity model that also values group differences but considers within-group differences to be of equal importance, thus ensuring that the individual is valued as well (Purdie-Vaughns & Walton, 2011; Steele et al., 2002; also see Gündemir, Homan, et al., 2017).

### Conclusion

As the world becomes increasingly diverse, efforts to harness the potential benefits of this diversity are essential. Although diversity efforts have become institutionalized in many companies and institutions, relatively limited research has examined the nuances of how to best manage diversity (Dobbin, 2009; Paluck, 2006), in a way that accounts for important individual differences in responses. A better understanding of both multicultural and colorblind practices will prevent unexpected consequences for underrepresented groups and help promote inclusion in the workplace.

## Supplemental Material

Kirby_and_Kaiser_2020_Supplemental_Material – Supplemental material for Person-Message Fit: Racial Identification Moderates the Benefits of Multicultural and Colorblind Diversity Approaches Click here for additional data file. Supplemental material, Kirby_and_Kaiser_2020_Supplemental_Material for Person-Message Fit: Racial Identification Moderates the Benefits of Multicultural and Colorblind Diversity Approaches by Teri A. Kirby and Cheryl R. Kaiser in Personality and Social Psychology Bulletin

## Notes

1. Colorblindness has been defined in a variety of ways, including a focus on individual differences or uniqueness (Gündemir, Dovidio, et al., 2017; Rosenthal & Levy, 2010) or on equality (Apfelbaum et al., 2016), but we will focus on downplaying as opposed to accentuating differences.

2. Some scholars have defined multiculturalism as an approach that values diverse backgrounds (e.g., Berry, 2001) and colorblindness as one that devalues diversity, perhaps even ignoring race and ethnicity as a category altogether (e.g., Apfelbaum et al., 2008; Norton et al., 2006). However, this definition can create a valence or prejudice confound (Hahn et al., 2015). It is important to distinguish between the goal of valuing diverse identities as opposed to the approach prescribed for navigating difference (Hahn et al., 2015), and the latter is our focus. For example, a society or an organization can express a commitment to diversity, while still advocating a colorblind approach that downplays intergroup difference for the sake of harmony. Indeed, a large majority of organizations in the United States express a commitment to diversity (Dobbin, 2009; Kalev et al., 2006), but downplaying difference is nonetheless a common approach (Plaut, 2002), suggesting that these ideas can exist alongside each other. In a great deal of research, the prescription to emphasize versus downplay group differences is one of the essential components distinguishing multiculturalism versus colorblindness (Gündemir et al., 2019) and is the approach we will take in the present research.

3. We will henceforth use the term *identity* to refer to group-based identities, as group identification is the focus of this research. Although the individual self is also an aspect of identity, we will only use the term *individual self* in referring to this construct to draw a clear distinction.

4. The multicultural condition used by Purdie-Vaughns and colleagues (2008) read, “we believe that embracing our diversity enriches our culture,” but we adjusted the manipulation to, “we train our ethnically diverse workforce to embrace their differences.” This adjustment made it more parallel to the colorblind condition (“we train our ethnically diverse workforce to embrace their similarities”), which reflected the present study’s focus on group differences as opposed to similarities. We also focused exclusively on racial, ethnic, and cultural differences in the manipulation, rather than diversity more broadly, to avoid a possible conceptual overlap with diversity approaches that celebrate individual differences and uniqueness (Gündemir, Dovidio, et al., 2017).

5. An exploratory factor analysis using varimax rotation with maximum likelihood estimation showed that anxiety, authenticity, and prototypicality pressure loaded onto three separate factors. Full statistical analyses are available in Supplemental Table S2.

6. To ensure the coefficients are comparable, this approach requires using the same independent variables across all regressions. Because one study did not have a control group, we first ran regressions to obtain coefficients for the multiculturalism versus colorblind comparison, leaving out the control groups for all experiments. Next, we ran the usual regression models for comparisons with the control group in Experiments 1, 3, 4, and 5.

7. Two research assistants also independently coded the stereotypicality (intraclass correlation coefficient [ICC] = .80) of each essay to use as a covariate in analyses. There were no effects of condition or interaction between condition and racial identification on stereotypicality of essays, *p*s > .125. In addition, when controlling for stereotypicality in analyses of hiring desirability, authenticity, and anxiety, all previously significant *p* values remained statistically significant, suggesting that judgments of authenticity and anxiety were not merely capturing stereotypicality.
