# Supplementary material for: Person-Message Fit: Racial Identification Moderates the Benefits of Multicultural and Colorblind Diversity Approaches
Source: Pers Soc Psychol Bull. 2020 Sep 15;47(6):873–90. doi: 10.1177/0146167220948707 (PMC8107502; doi:10.1177/0146167220948707)
Supplement: Kirby_and_Kaiser_2020_Supplemental_Material – Supplemental material for Person-Message Fit: Racial Identification Moderates the Benefits of Multicultural and Colorblind Diversity Approaches [file Kirby_and_Kaiser_2020_Supplemental_Material.pdf]

Person-Message Fit: Racial Identification Moderates the Benefits of Multicultural and Colorblind Diversity Approaches

Online Supplement

Teri A. Kirby
University of Exeter
Cheryl R. Kaiser
University of Washington

Contents

Sensitivity Analyses 2
Experimental Manipulations 3
Measures 9
Full Details of Experiment 1 12
Method 12
Results 13
Full Details of Experiment 2 20
Method 20
Results 24
Full Details of Experiment 3 30
Method 30
Results 35
Full Details of Experiment 4 37
Method 37
Results 40
Full Details of Experiment 5 45
Method 45
Results 49
Stereotype Pilot Studies 58
Activity Stereotyping Pilot Study 58
Trait Stereotype Testing 60

Sensitivity Analyses

Table S1. Information for sensitivity calculations across all studies.

	Study				
	1	2	3	4	5
*N*s per condition (MC, CB)	95, 88	-	122, 109	126, 130	66, 55
SD IV (MC, CB)	1.54, 1.50	-	1.49, 1.59	1.51, 1.66	1.39, 1.53
Authenticity $SD\ E_{ij}$	1.37	-	1.19	1.22	0.60
Anxiety $SD\ E_{ij}$	1.53	-	1.72	1.45	0.57
**Authenticity Sensitivity β**	**0.38**	**-**	**0.29**	**0.27**	**0.21**
**Anxiety Sensitivity β**	**0.42**	**-**	**0.41**	**0.32**	**0.20**

Note. The sensitivity analysis section gives the information to calculate the minimum effect (β) needed to detect a difference in the multicultural (MC) and colorblind (CB) slopes (i.e., an interaction between racial identification and condition). This information is presented for the two key variables, authenticity and anxiety, with 80% power at $\alpha = .05$.The sensitivity analysis requires entering the standard deviation of the error term ($SD\ E_{ij}$) and moderator variable (*SD* IV: racial identification), as well as *n* per condition, all included in the table above.

Experimental Manipulations

Experiments 1-3, 5

Colorblind brochure

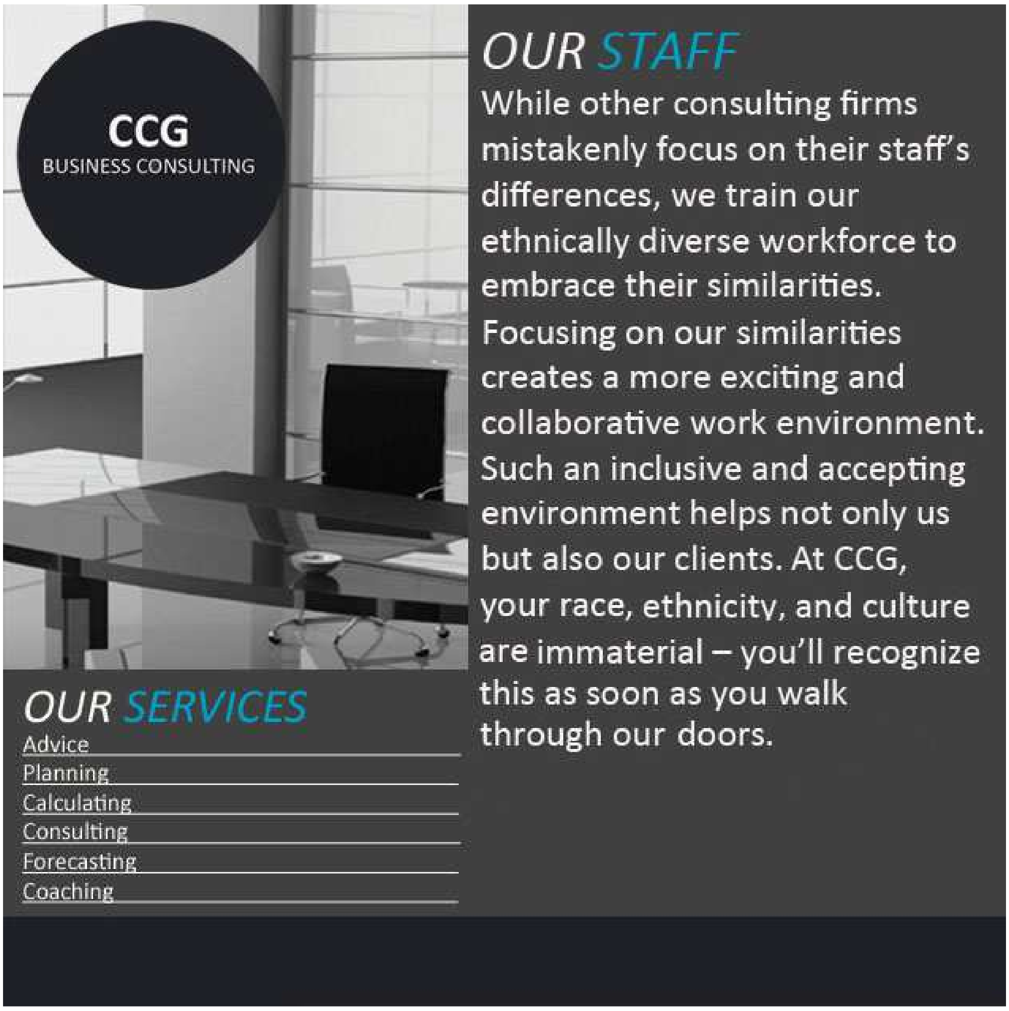

Multicultural brochure

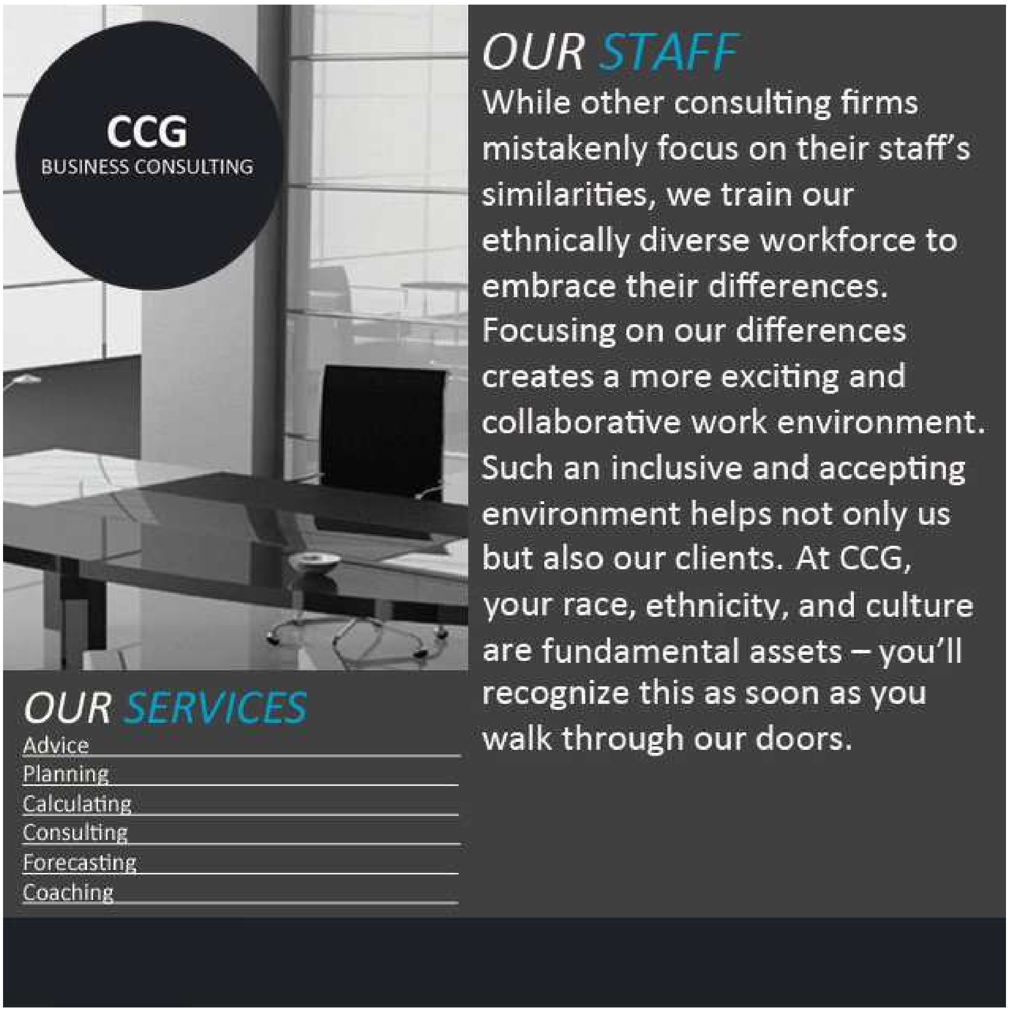

Control brochure (Experiments 2 & 3)

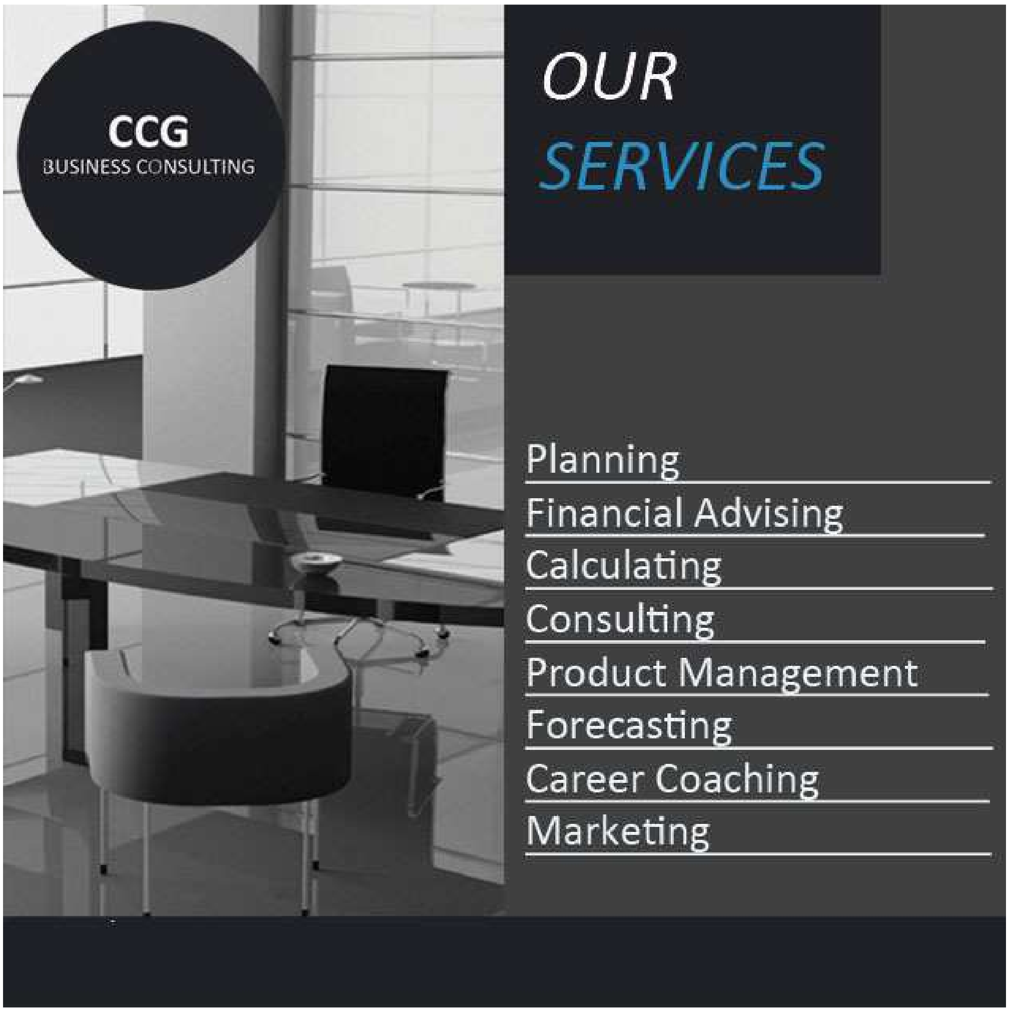

Experiment 3

Colorblind brochure

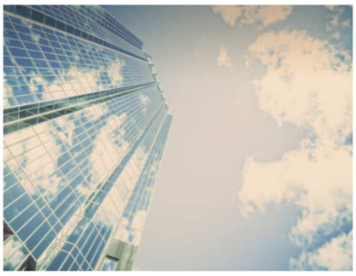

Who We Are

CCG is a major provider of consultancy services, with a reputation for excellence. With annual turnover of $300 million, the company places a strong emphasis on business growth and the contribution of its staff.

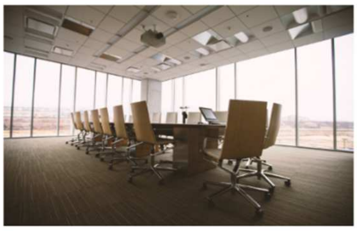

Our Staff

While other consulting firms mistakenly focus on their staff's differences, we train our ethnically diverse workforce to embrace their similarities.

Focusing on our similarities creates a more exciting and collaborative work environment. Such an inclusive and accepting environment helps not only us, but also our clients.

At CCG, your race, ethnicity, and culture are immaterial—you'll recognize this as soon as you walk through our doors.

|CCG

Our Mission

We aim to ensure that working with CCG is a smooth and effective process for our clients through:

- Excellence and expertise in quality of services
- Customer-focused culture
- Continual process improvement
- Success in implementing pioneering activities and technology

Contact Us

CCG
45 West 51st Street. 28th Floor.

New York, NY 11020

1 (212) 566 9000
CCG_Inquiries@ccg.com

Multicultural brochure

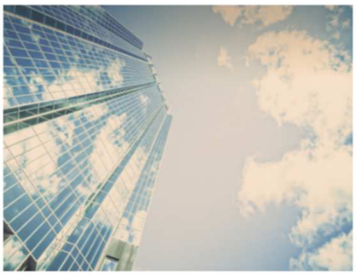

Who We Are

CCG is a major provider of consultancy services, with a reputation for excellence. With annual turnover of $300 million, the company places a strong emphasis on business growth and the contribution of its staff.

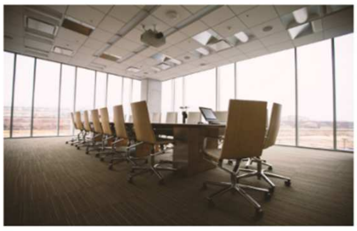

Our Staff

While other consulting firms mistakenly focus on their staff's similarities, we train our ethnically diverse workforce to embrace their differences.

Focusing on our differences creates a more exciting and collaborative work environment. Such an inclusive and accepting environment helps not only us but also our clients.

At CCG, your race, ethnicity, and culture are fundamental assets—you'll recognize this as soon as you walk through our doors.

CCG

Our Mission

We aim to ensure that working with CCG is a smooth and effective process for our clients through:

- Excellence and expertise in quality of services
- Customer-focused culture
- Continual process improvement
- Success in implementing pioneering activities and technology

Contact Us

CCG
45 West 51st Street. 28th Floor.

New York, NY 11020

1 (212) 566 9000
CCG_Inquiries@ccg.com

Control brochure

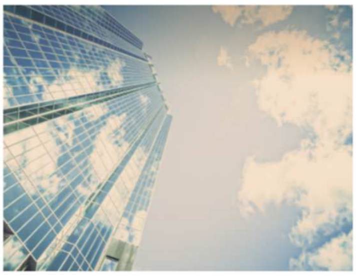

Who We Are

CCG is a major provider of consultancy services, with a reputation for excellence. With annual turnover of $300 million, the company places a strong emphasis on business growth and the contribution of its staff.

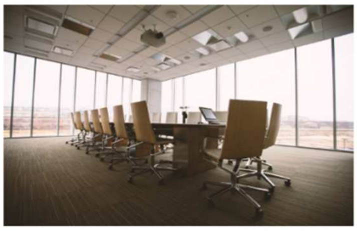

Our Staff

At CCG, we seek to foster an environment that focuses on our staff—this creates a more exciting work environment.

This environment helps not only us, but our clients as well. At CCG, our staff has unlimited access to success.

CCG

Our Mission

We aim to ensure that working with CCG is a smooth and effective process for our clients through:

- Excellence and expertise in quality of services
- Customer-focused culture
- Continual process improvement
- Success in implementing pioneering activities and technology

Contact Us

CCG
45 West 51st Street. 28th Floor.

New York, NY 11020

1 (212) 566 9000
CCG_Inquiries@ccg.com

Measures

Racial Identification (All experiments)

My race/ethnicity is unimportant to my sense of what kind of a person I am. (reverse scored)
The racial/ethnic group I belong to is an important reflection of who I am.
In general, belonging to my race/ethnicity is an important part of my self image.
Overall, my race/ethnicity has very little to do with how I feel about myself. (reverse scored)

1 = *Strongly disagree*, 2 = *Disagree*, 3 = D*isagree somewhat*, 4 = *Neutral*, 5 = *Agree somewhat*, 6 = *Agree*, 7 = *Strongly agree*

Prototypicality Pressure (Experiment 1)

CCG would be more likely to hire me if I asserted my racial/ethnic identity.
My interview at CCG would go better if I asserted my racial/ethnic identity.
If I asserted my racial/ethnic identity, CCG would think I was a better fit at their company.
CCG would be more likely to hire me if I conformed to their expectations about my racial/ethnic group.
My interview at CCG would go better if I behaved like a representative of my racial group.
If I seemed like others of my racial group, CCG would think I was a better fit at their company.

1 = *Strongly disagree*, 2 = *Disagree*, 3 = *Moderately disagree*, 4 = *Neutral*, 5 = *Moderately agree*, 6 = *Agree*, 7 = *Strongly agree*

Authenticity (Experiments 1,3,4,5)

I would be myself at the CCG interview.
I would be my true self at the CCG interview.
I would feel comfortable being myself at the CCG interview.
I would feel comfortable at the CCG interview.

Anxiety (Experiment 1,3,4,5)

I would feel anxious at the CCG interview.
I would feel nervous at the CCG interview.
I would feel uncomfortable at the CCG interview. *[This item also loaded onto the authenticity scale, but is included only in the anxiety scale because it loaded slightly more strongly and boosted the reliability of this scale]*

1 = *Strongly disagree*, 2 = *Disagree*, 3 = *Moderately disagree*, 4 = *Neutral*, 5 = *Moderately agree*, 6 = *Agree*, 7 = *Strongly agree*

Workplace Citizenship (Experiments 3-4)

In past jobs, how often have you helped other colleagues?
In past jobs, how often have you volunteered to help with company activities?
In past jobs, how often have you worked extra hours?
In past jobs, how often have you gone above and beyond what a task required?

1 = *Never*, 2 = *Occasionally*, 3 = *Sometimes*, 4 = *Often*, 5 = *All the time*

Experiment 5 Essay Prompt

[Instructions Page 1]

Section 1/5

Please imagine that you are interviewing at CCG, and they would like to know more about you as a person.

CCG offers a number of benefits to its employees such as museum and gym memberships, discounted concerts, sporting events, theater tickets, and many other activities. It also organizes events for employees and friends/family. Your responses to these questions may inform the type of benefits that Human Resources offers in the future or the types of events it organizes. Please keep this in mind when responding to the next question.

Click to Continue

[Instructions Page 2]

CCG Questionnaire

Please spend a couple minutes answering the following question. Please be detailed and write as much as you want (although it is not required, normal responses are at least five lines). All of the questions that follow this one are multiple choice.

If you had to describe yourself to someone at CCG, how would you describe yourself? For example, what are some of your favorite activities, hobbies, and interests? What are your favorite academic subjects or other educational pursuits? Who do you enjoy spending time with, and where? How would you describe your personality? Please be as specific as possible (for example, if you enjoy music, what type of music?).

Full Details of Experiment 1

Below, we describe individual study results that were not reported in the main text due to space constraints or that were only reported in aggregate in the main text (e.g., simple effects for interactions).

Method

Participants did not complete explicit self-stereotyping measures in this study due to limitations on study length and because understanding self-stereotyping was not a central aim in this study; however, they completed an implicit self-stereotyping measure because Project Implicit volunteers visit the website to learn about their implicit attitudes. The implicit measure allowed us to ascertain whether authenticity concerns would lead participants to adjust their self-stereotyping and whether it reflected an automatic or a more deliberate process (see Cvencek, Greenwald, Brown, Gray, & Snowden, 2010; also Gawronski, LeBel, & Peters, 2007).

The implicit measure was adapted from Amodio and Devine's (2006) Brief Implicit Association Test (BIAT) assessing stereotypes of African Americans as more athletic (physical) and less intelligent (mental) relative to European Americans (Devine & Elliot, 1995). In six blocks (alternating two different block types), participants classified “mental” words (e.g., math, brainy), “physical” words (e.g., athletic, dancing), “self” words (e.g., me, self), and “other” (e.g., other, they) words using two response keys. In one block type, they pressed the right key for mental and self words (and the left key for all other words); in the second block type, they pressed the right key for physical and self words. Which block type participants completed first was counter-balanced. Participants classifying physical and self words together more quickly than mental and self words indicated a stronger automatic association of themselves with physical than mental activities. The BIAT was scored using

the IAT *D* measure (Greenwald, Nosek, & Banaji, 2003) so that positive values corresponded to greater implicit self-association with physical activities.

Results

Manipulation and attrition checks. Perceptions of how much the company focused on group differences differed by condition, $F(2, 242) = 37.17$, $p < .001$. Specifically, Bonferroni multiple comparison tests revealed that participants perceived greater focus on group differences in the multiculturalism condition ($M = 3.27$, $SD = 0.98$) than in the colorblind condition ($M = 2.05$, $SD = 1.12$), $p < .001$, and the control condition ($M = 2.16$, $SD = 0.99$), $p < .001$. However, participants did not perceive a difference in how much the control and colorblind companies focused on group differences, $p = .775$. Attrition from the study did not differ by gender, $\chi^2(1, n = 403) = 1.14$, $p = .286$, but did differ by diversity condition, $\chi^2(2, n = 403) = 11.57$, $p = .003$. Participants assigned to the control condition completed the study at lower rates (53%) than those assigned to the multicultural (73%) or colorblind (66%) conditions.

Preliminary analyses. To confirm that the prototypicality pressure, authenticity, and anxiety items loaded onto the anticipated measures, we conducted a factor analysis with maximum likelihood estimation requesting three factors. See Table S2 for results and Table S3 for correlations between aggregated measures.

Table S2

Factor Analysis on Dependent Variable Items in Experiment 1

Items	Factor Loadings		
	Prototypicality Pressure	Authenticity	Anxiety
CCG would be more likely to hire me if I conformed to their expectations about my racial ethnic group	.36		
CCG would be more likely to hire me if I asserted my racial ethnic identity	.84		
If I asserted my racial ethnic identity, CCG would think I was a better fit at their company	.84		
My interview at CCG would go better if I asserted my racial ethnic identity	.84		
If I seemed like others of my racial group, CCG would think I was a better fit at their company	.52		
My interview at CCG would go better if I behaved like a representative of my racial group	.50		
I would feel nervous at the CCG interview			.82
I would feel uncomfortable at the CCG interview		.44	.53
I would feel anxious at the CCG interview			.74
I would be myself at the CCG interview (reverse)		.69	
I would feel comfortable being myself at the CCG interview (reverse)		.91	
I would feel comfortable at the CCG interview (reverse)		.62	.45
I would be my true self at the CCG interview (reverse)		.68	

Note. Loadings below .30 are suppressed for clarity of presentation. For items that loaded onto more than one factor, we included them in the scale where they had the strongest loading.

Table S3

Means, Standard Deviations, and Correlations Between Primary Experiment 1 Variables

	1	2	3	4
1. Racial Identification	-			
2. Prototypicality Pressure	-.04	-		
3. Authenticity	.03	$-.19^{**}$	-	
4. Anxiety	.01	$.17^{**}$	$-.50^{***}$	-
Mean (*SD*)	4.59 (1.50)	3.43 (1.24)	5.26 (1.32)	3.81 (1.48)

Note. *N* = 256. Numbers in parentheses next to means correspond to standard deviations. Scales range from 1-7 for all measures.

$p < .05$ ** $p < .01$ *** $p < .001$

Main analyses. As described in the main text, we probed the highest order significant interactions with simple effects and then with simple slope analyses using the PROCESS macro (Hayes, 2013). We defined "strong" and "weak" racial identification, respectively, as those who moderately agreed (6 on a 7 point scale) and moderately disagreed (2 on a 7 point scale).

Authenticity. The predicted two-way interaction (ΔR^2 = .04, *p* = .010) emerged between the multiculturalism condition (relative to colorblindness) and racial identification, β = -0.26, *t*(250) = -3.05, *p* = .003, but not for the multiculturalism condition (relative to control) and racial identification, β = -0.08, *t*(250) = -1.01, *p* = .315[1]. Next, we examined simple effects for the multicultural relative to colorblind comparison.

In simple effects analyses, consistent with predictions, African Americans with weak racial identification reported that they would feel less comfortable being authentic when exposed to a company that valued multiculturalism relative to colorblindness, *b* = 1.06, *SE* = 0.38, *p* = .006.African Americans with strong racial identification reported that they would feel more comfortable being authentic when exposed to a company that valued multiculturalism relative to colorblindness, *b* = -0.50, *SE* = 0.27, *p* = .060, but it did not reach conventional levels of statistical significance at the value designated for high racial identification.

Simple slope analyses showed the predicted interaction pattern as well. As participants in the colorblind condition were more strongly racially identified, they felt less comfortable being authentic, *b* = -0.20, *SE* = 0.09, *p* = .035. In the multiculturalism condition, as participants were more strongly racially identified, they instead felt more comfortable

[1] We also re-ran the regression model with the control condition as the reference group to compare the control and colorblind conditions. There was no interaction between the colorblind condition (relative to control) and racial identification, β = -0.17, *t*(250) = -1.79, *p* = .075.

being authentic, *b* = 0.19, *SE* = 0.09, *p* = .028. Finally, the relationship between racial identification and authenticity was not statistically significant in the control condition, *b* = 0.05, *SE* = 0.11, *p* = .606.

Anxiety. The predicted two-way interaction ($\Delta R^2 = .03$, *p* = .012) emerged between the multiculturalism condition (relative to colorblindness) and racial identification, β = 0.23, *t*(250) = 2.73, *p* = .007, and the multiculturalism condition (relative to control) and racial identification, β = 0.19, *t*(250) = 2.32, *p* = .021. [2] Next, we examined simple effects for both the multicultural relative to colorblind and the multicultural relative to control comparisons.

In simple effects analyses, consistent with predictions, African Americans with weak racial identification reported that they would feel more anxious when exposed to a company that valued multiculturalism relative to colorblindness, *b* = -1.15, *SE* = 0.43, *p* = .008, but not relative to the control condition, *b* = -0.62, *SE* = 0.46, *p* = .149. African Americans with strong racial identification showed the opposite pattern, but this difference did not reach conventional levels of statistical significance for the multicultural relative to colorblind comparison, *b* = 0.41, *SE* = 0.30, *p* = .168, although it did for the multicultural relative to control comparison, *b* = 0.81, *SE* = 0.31, *p* = .010.

Simple slope analyses partially confirmed predictions in terms of the slope patterns, but some of the slopes did not reach conventional levels of statistical significance. In the multiculturalism condition, as participants were stronger in racial identification, they were less anxious, *b* = -0.22, *SE* = .10, *p* = .028. The relationship was the reverse (but non-significant) in the control condition, *b* = 0.14, *SE* = 0.12, *p* = .239, and the colorblind condition *b* = 0.17, *SE* = .10, *p* = .098.

[2] We also re-ran the regression model with the control condition as the reference group to compare the control and colorblind conditions. There was no interaction between the colorblind condition (relative to control) and racial identification, β = .020, *t*(250) = 0.21, *p* = .832.

Prototypicality pressure mediating authenticity and anxiety. We tested moderated mediation models with 10,000 bootstrap resamples using the PROCESS macro (Model 15; Hayes, 2013) to determine whether prototypicality pressure statistically mediated the effect of multiculturalism (relative to colorblindness) on (1) authenticity and (2) anxiety among weakly racially identified participants (but not strongly identified). In the full model for authenticity (1), the index of moderated mediation was significant for both the multiculturalism compared to colorblind, b = -0.09, SE = 0.04, 95% CI[-0.18, -0.01], and control comparisons, b = -0.09, SE = 0.04, 95% CI[-0.18, -0.01]. When examining mediation by prototypicality pressure at different levels of the racial identification moderator, indeed, there was a significant indirect effect on authenticity among those weakly identified for the colorblind (v multiculturalism) comparison, b = 0.29, SE = 0.10, 95% CI[0.11, 0.49], and for the control (v multiculturalism) comparison, b = 0.29, SE = 0.10, 95% CI[0.11, 0.50]. There was not a significant indirect effect among those strongly identified for the colorblind (v multiculturalism) comparison, b = 0.03, SE = 0.09, 95% CI[-0.15, 0.20] or for the control (v multiculturalism) comparison, b = 0.03, SE = 0.09, 95% CI[-0.15, 0.20].

In the full model for anxiety (2), however, the index of moderated mediation was not significant for either the multiculturalism compared to colorblind, b = 0.04, SE = 0.05, 95% CI[-0.04, 0.14], or control comparisons, b = 0.04, SE = 0.05, 95% CI[-0.04, 0.14]

Implicit self-stereotyping. As shown in Table S4, there were no effects of diversity condition on implicit self-stereotyping.

Table S4

Hierarchical Regression on Implicit Self-Stereotyping in Experiment 1

Predictor	β	*p*
Step 1	$\Delta R^2 = .01$, $p = .614$	
Racial Identification	0.07	.337
Control (v. Multicultural)	0.07	.363
Colorblind (v. Multicultural)	0.03	.672
Step 2	$\Delta R^2 < .001$, $p = .968$	
Control (v. Multicultural) x Identification	0.01	.920
Colorblind (v. Multicultural) x Identification	0.03	.800

Note. Regression coefficients are reported from the step on which each variable was first entered. The multicultural condition, the reference group in the regression, is always coded as 0, with control and colorblindness coded as 1.

Full Details of Experiment 2

Method

Participants. 2,537 White and 337 African American visitors to the Project Implicit website (https://implicit.harvard.edu), who volunteered to participate in implicit social cognition research, were randomly assigned to complete the present study from a pool of available studies (previous participants were not permitted to participate). Sixty-six participants were excluded because they indicated in an open-ended item that they did not read the brochure containing the manipulation, and another 135 were excluded because 10% or more of their BIAT trials were faster than 300 milliseconds (as recommended by Greenwald, Nosek, & Banaji, 2003)[3] or for nonsensical questionnaire responses (e.g., reporting the same response for all items, including reverse-scored ones). Of the remaining 1,623 participants, 1,487 White (1,109 women, 377 men) and 136 African Americans (95 women, 41 men) reached the end of the study and completed the key independent and dependent measures (mean age = 33.38, *SD* = 11.29; 98% had completed some college or a higher level of education). To maximize statistical power, we retained partial data for those not fully completing the study, resulting in varying degrees of freedom in analyses (as in all subsequent studies). For African Americans, accounting for attrition, this left us with adequate power (π = .80) to detect a slope difference by condition (i.e., an interaction between racial identification and condition) of β = 0.49 for self-stereotyping. Our goal sample size was 70 per condition.

Additional Information about Measures

[3] Results were the same when analyzing without exclusions.

African American negative trait stereotypes. In addition to the positive trait measure described in the main text[4], participants responded about the extent to which eight negative stereotypes of African Americans described them (poor, lazy, reckless, dishonest, dangerous, complaining, violent, ignorant; α= .73; Judd, Park, Ryan, Brauer, & Kraus, 1995; Wolsko, Park, Judd, & Wittenbrink, 2000). Scale endpoints were 1 (*Not at all descriptive of me*) to 7 (*Very descriptive of me*).

White trait stereotypes. Participants also responded about the extent to which six positive stereotypes (wealthy, responsible, successful, educated, intelligent, ambitious; α= .74; Judd, Park, Ryan, Brauer, & Kraus, 1995; Wolsko, Park, Judd, & Wittenbrink, 2000) and seven negative stereotypes (boring, materialistic, greedy, conventional, uptight, stuffy, boastful; α= .68) of Whites described them. Scale endpoints were 1 (*Not at all descriptive of me*) to 7 (*Very descriptive of me*). The inclusion of White traits allowed us to determine whether diversity philosophies also affected Whites' self-stereotyping and whether diversity philosophies affected African Americans' self-descriptions only on measures relevant to stereotypes of their group (i.e., African American stereotypes) or on all types of traits, irrespective of the association of the traits with African Americans (i.e., White American stereotypes).

Racial identification. Participants responded to the same racial identification scale as in Experiment 1 (α = .76). African American participants (M = 4.44, SD = 1.47) reported stronger racial identification than White participants (M = 3.27, SD = 1.31), $F(1,1621)$ = 9.94,

[4] A preliminary study revealed an unexpected gender moderation on self-stereotyping (with predicted effects demonstrated among men, but not women). However, we realized in retrospect that the traits and activities used in the preliminary study were stereotypically masculine ones and may not have captured stereotypes of African American women. Indeed, African American women are often overlooked in stereotype measurement because men are the prototype of their group (see Ghavami & Peplau, 2012; Purdie-Vaughns & Eibach, 2008; Sesko & Biernat, 2010). Accordingly, in this study, we ensured that measures also included female African American stereotypes (i.e., emotionally expressive, talking, gospel music; see the final section of the online supplement for more information about piloting).

p < .001, *d* = 0.85. Due to racial differences in identification, we mean centered racial identification by race before including it in the main regression analyses.

The centrality dimension of racial identification is theorized to be stable across situations (Sellers et al., 1998), and participants' level of racial identification did not differ across conditions, *F*(1,1619) = 1.22, *p* = .271, nor was the effect of condition moderated by race, *F*(1,1619) = .001, *p* = .979.

Manipulation check. To determine whether participants interpreted the manipulation as intended, they responded to the following item: "To what extent does CCG value group differences?" on a 1 (*Undervalue a great deal*) to 7 (*Value a great deal*) scale.

Table S5

Means, Standard Deviations, and Correlations Between Primary Experiment 2 Variables Split by Participant Race

	1	2	3	4	5	6
1. Racial Identification	-	.04 (1487)	.003 (1487)	.01 (1486)	.003 (1487)	.05 (1486) $^{+}$
2. Black Activity Stereotypes	.03 (136)	-	.37 (1487)***	-.003 (1486)	.21 (1487)***	<-.001 (1486)
3. Positive Black Trait Stereotypes	-.01 (136)	.54 (136)***	-	.04 (1486)	.45 (1487)***	-.13 (1486)***
4. Negative Black Trait Stereotypes	.01 (136)	-.001 (136)	.23 (136)**	-	-.27 (1486)***	.53 (1486)***
5. Positive White Trait Stereotypes	-.01 (136)	.25 (136)**	.32 (136)***	-.28 (136)***	-	-.07 (1486)**
6. Negative White Trait Stereotypes	.07 (136)	.02 (136)	.19 (136)*	.67 (136)***	-.02 (136)	-
Mean (*SD*) for African Americans	4.44 (1.47)	4.02 (1.25)	4.07 (1.23)	1.72 (0.73)	5.22 (0.95)	2.39 (0.75)
Mean (*SD*) for Whites	3.27 (1.31)	3.25 (0.96)	3.80 (1.01)	1.95 (0.68)	4.89 (0.86)	2.66 (0.82)

Note. Correlations for African Americans are below the diagonal, and correlations for Whites are above the diagonal. *N*s (in parentheses next to correlations) do not necessarily correspond to the degrees of freedom in regression analyses, as we used pairwise deletion for all analyses. Numbers in parentheses next to means correspond to standard deviations. Scales range from 1-7 for all measures.
* $p < .05$ ** $p < .01$ *** $p < .001$

Supplementary dependent measures. The primary purpose of the research was to understand how diversity approaches shape self-views in the organizational context. However, we included two additional measures described below that assessed participants' perceptions of how they would be treated and fit at the company.

Company stereotyping. This exploratory dependent variable consisted of two items ("People at this company would stereotype your racial group"; "People at CCG would treat you differently based on your racial group membership"; ρ = .88) on a 1 (Strongly Disagree) to 7 (Strongly Agree) scale. We used the Spearman-Brown formula, recommended by Eisinga, Grotenhuis, and Pelzer (2013) to calculate reliability with two-item measures.

Change to fit in. This variable was measured with a single item ("I would have to change to fit in at this company") on a 1 (Strongly Disagree) to 7 (Strongly Agree) scale.

Results

Manipulation and attrition checks. Participants reported that the company valued group differences more in the multiculturalism condition (M = 6.46, SD = 1.10) than in the colorblind condition (M = 3.22, SD = 2.31), $F(1, 1451) = 216.81$, $p < .001$, d = 1.94. This effect was more pronounced among White participants (multicultural M = 6.51; colorblind M = 3.13), $F(1, 1451) = 1206.15$, $p < .001$, than among African American participants (multicultural M = 5.94; colorblind M = 4.22), $F(1, 1451) = 26.94$, $p < .001$; interaction: $F(1,1451) = 22.67$, $p < .001$.

Attrition from the study did not differ by diversity condition, $\chi^2(1, n = 2674) = 1.56$, $p = .212$, or gender, $\chi^2(1, n = 2669) = 1.34$, $p = .248$. However, White participants (62%) completed the study at higher rates than African Americans (47%), $\chi^2(1, n = 2674) = 25.26$, $p < .001$, but this effect did not interact with condition, $\chi^2(1, n = 2674) = 0.60$, $p = .438$.

Analytic strategy. To test the main hypotheses, diversity condition (0 = multiculturalism, 1 = colorblindness,), race (0 = African American, 1 = White), and racial identification (mean-centered by race) were entered into the first step of a hierarchical linear regression model. All two-way interactions were entered into the second step, and the single three-way interaction was entered into the third step. We followed up with simple slope and simple effects analyses for the highest order significant interactions (defined as $p < .05$ throughout all studies) using the PROCESS macro (Hayes, 2013). We conducted simple effects analyses as described in Experiment 1.

Main self-stereotyping analyses. Unless otherwise specified, we hypothesized an interaction between diversity condition, race, and racial identification such that African Americans, but not Whites, would show a two-way interaction between racial identification and diversity condition.

African American activity stereotypes. The predicted three-way interaction between diversity condition, racial identification, and race emerged, $\beta = -0.26$, $t(1615) = -2.37$, $p = .018$. We first broke down the three-way interaction by participant race. Consistent with hypotheses, the two-way interaction between condition and racial identification emerged among African American participants, $b = 0.27$, $p = .021$, but not White participants, $b = -0.02$, $SE = 0.04$, $p = .561$, so we did not examine Whites further.

In simple effects analyses, consistent with predictions, African American participants with weak racial identification reported more interest in stereotypically African American activities when exposed to a company that valued multiculturalism relative to colorblindness $b = -0.91$, $SE = 0.33$, $p = .006$. Strongly racially identified African American participants showed the opposite pattern, but this difference did not reach conventional levels of statistical significance, $b = 0.16$, $SE = 0.25$, $p = .515$.

Simple slope analyses confirmed the predicted interaction pattern as well. As African Americans in the colorblind condition had stronger racial identification, they self-stereotyped more on the activity measure, *b* = 0.17, *SE* = 0.09, *p* = .040. In the multiculturalism condition, this relationship was attenuated, and there was no relationship between racial identification and self-stereotyping, *b* = -0.09, *SE* = 0.08, *p* = .237.

African American positive trait stereotypes. The predicted three-way interaction between diversity condition, racial identification, and race once again emerged, β = -0.24, *t*(1615) = -2.12, *p* = .034. We first broke down the interaction by participant race. Consistent with hypotheses, a two-way interaction between condition and racial identification emerged among African American participants, *b* = 0.25, *p* = .035, but not White participants, *b* = -0.02, *p* = .708, so we did not examine Whites further.

In simple effects analyses, consistent with predictions, African Americans with weak racial identification self-stereotyped more on positive traits when exposed to a company that valued multiculturalism relative to colorblindness, *b* = -0.83, *SE* = 0.35, *p* = .017. Strongly racially identified African American participants showed the opposite pattern, but this difference did not reach conventional levels of statistical significance, *b* = 0.19, *SE* = 0.26, *p* = .454.

Simple slope analyses showed the predicted interaction pattern, but the slopes were not statistically significant for either condition. We nonetheless describe the pattern of these results to facilitate understanding of the significant interaction terms. As African Americans in the colorblind condition had stronger racial identification, there was a non-significant pattern of increased self-stereotyping, *b* = 0.13, *SE* = 0.09, *p* = .141. In the multiculturalism condition, this relationship was attenuated, and the pattern was in the opposite direction, *b* = -0.12, *SE* = 0.08, *p* = .129.

African American negative trait stereotypes. Because people tend to embrace positive, but not negative, stereotypes about their group (i.e., selective self-stereotyping; Biernat, Vescio, & Green, 1996), and may be particularly unwilling to express negative stereotypes in the context of job outcomes, we did not expect diversity condition to affect levels of negative self-stereotyping. Indeed, participants were relatively unwilling to express negative stereotypes about themselves, resulting in a positive skew on this variable (skewness = 0.89, $SE = .06$; $M = 1.94$, $SD = 0.69$). Due to the low variability and because transformations of the variable did not reduce the skewness, it is difficult to interpret the results for negative stereotypically African American traits and we do not describe these results in detail (however, no main effects or interactions emerged for any variables of theoretical interest, ps > .176).

Implicit stereotypes. Consistent with Study 1, there were no main effects or interactions with diversity condition, ps > .354.

Analyses of White trait stereotypes. We hypothesized that neither African Americans nor Whites would show an effect of diversity condition or an interaction between diversity condition and racial identification on measures reflecting stereotypes of Whites. If an effect emerged, however, this would suggest that diversity condition affects self-descriptions more broadly and not just traits and activities relevant to participants' group membership. As hypothesized, there were no main effects or interactions with diversity condition on positive traits, ps > .281, or negative traits, ps > .155, indicating that diversity condition only affected descriptions with stereotype-relevant traits among African Americans.

Supplementary dependent measures. The results presented in Table S6 conceptually replicated findings by Purdie-Vaughns, Steele, Davies, Ditlmann, and Crosby (2008) suggesting that racial minorities trust multicultural more than colorblind companies.

Specifically, African Americans believed that people at the colorblind company would stereotype them more than those at the multicultural company, although the reverse was true for Whites. Additionally, both Whites and African Americans believed they would have to change to fit in more at the colorblind than multicultural company. This was magnified for those strongly identified with their racial group.

Table S6

Hierarchical Regression on Supplemental Dependent Variables in Experiment 2

	Company Stereotyping		Change to Fit In	
Predictor	β	*p*	β	*p*
Step 1	$\Delta R^2 = 0.03$, $p < .001$		$\Delta R^2 = 0.04$, $p < .001$	
Diversity Condition	-0.11	<.001	0.16	< .001
Racial Identification	0.13	<.001	0.11	< .001
Race	-0.08	.001	0.05	.031
Step 2	$\Delta R^2 = 0.01$, $p < .001$		$\Delta R^2 = 0.01$, $p = .010$	
Diversity x Centrality	0.06	.120	0.11	.002
Diversity x Race	-0.35	<.001	-0.11	.226
Race x Centrality	0.08	.287	-0.02	.846
Step 3	$\Delta R^2 < 0.001$, $p = .655$		$\Delta R^2 = 0.001$, $p = .242$	
Diversity x Race x Centrality	-0.05	.655	-0.13	.242

Note. Regression coefficients are reported from the step on which each variable was first entered. For diversity condition, 0 =Multiculturalism, 1 = Colorblindness. For Race, 0 = African American, 1 = White.

Full Details of Experiment 3

This experiment was pre-registered at https://osf.io/2j4aw.

Method

Participants. We recruited Amazon Mechanical Turk workers through Turkprime, an online crowdsourcing platform that allows for recruitment of participants with specified demographic criteria (see Litman, Robinson, & Abberbock, 2017, for more information). In Wave 1, 604 African American participants completed racial identification measures and demographic information in exchange for US$0.30. Of these, 358 participated in Wave 2 (59%) for US $1.75, but 6 were excluded for nonsensical questionnaire responses (e.g., reporting the same response for all items) and failing an attention check. Of the remaining 352 participants (mean age = 35.86, *SD* = 11.29; 88% had completed some college or a higher level of education), 239 were women, 111 were men, 1 identified as another gender, and 1 gave no information. Everyone who started Wave 2 completed the study, and attrition between Wave 1 and Wave 2 of the study did not differ by gender, $\chi^2(2, n = 597) = 0.81, p = .667$, or racial identification, $F(1, 596) = 0.01, p = .921$.

We used G*power 3.1.5 to estimate the goal sample size in a linear bivariate regression (two groups, differences between slopes) with 80% power, an alpha level of 0.05, and an estimated slope difference of β = .394 between the multiculturalism and colorblind condition. Based on this, we aimed to collect at least 291 participants (97 per condition), but allowed as many participants as opted to participate in Wave 2.

Procedure. In Wave 1, participants completed the same four-item measure of racial identification described in previous studies, as well as demographic information. After an average of 26 days (*SD* = 18.24), participants were invited to participate in an ostensibly unrelated study (Wave 2). They were randomly assigned to read the same multiculturalism or colorblind philosophy in a CCG recruitment brochure from previous Experiments (see

Appendix B for new filler information and design) or a control statement that described CCG's staff philosophy without any reference to diversity. They next imagined that they were interviewing at CCG and completed the activity and positive trait self-stereotyping measures from Experiment 2 (in a random order), but with additional items (italicized) to boost reliability. Participants indicated the extent to which positive stereotypes of African Americans (streetwise, athletic, humorous, musical, emotionally expressive, *religious*, *rhythmic*, *spiritual*, *sporty*; $\alpha = .78$) were self-descriptive and how interested they were in several activities (rap music, gospel music, talking/socializing, sports/fitness, athletics, track, physical education, basketball, *attending religious services*, *singing*; $\alpha = .73$).

Next, participants responded to measures assessing workplace citizenship, interest in a race-relevant sub-organization, anticipated authenticity ($\alpha = .89$) and anxiety ($\alpha = .81$) in the interview, race concerns, state self-esteem, and racial identification, in that order. Finally, participants completed the same manipulation check item as in Experiment 2. Measures not already described in previous experiments are outlined below.

Additional information about measures

Workplace citizenship. We measured participants' reports of their past workplace citizenship in order to address an alternative explanation for self-stereotyping. Although we hypothesized that weakly identified participants would self-stereotype more in the multicultural than colorblind condition, self-stereotyping might have picked up more generally on positive self-views or self-presentation. If weakly identified minorities described their workplace citizenship histories more positively in the multicultural company, this would be consistent with the positive self-presentation alternative explanation (and inconsistent with hypotheses). Specifically, participants responded to four items (see Appendix C; e.g., "In past jobs, how often have you helped other colleagues?"; "In past jobs, how often have you worked extra hours") on a 1 (*Strongly disagree*) to 7 (*Strongly agree*) scale; $\alpha = .82$.

Race concerns. Participants responded to two items ("I would be worried about whether I conform to CCG's expectations for my racial/ethnic group" and "I would be concerned about whether I am a good representative of my racial/ethnic group") on a 1 (*Strongly disagree*) to 7 (*Strongly agree*) scale; $\rho = .80$.

State self-esteem. To assess participants' self-views, they responded to 10 items about how they would feel right now if they worked at CCG (e.g., "I take a positive attitude toward myself right now") from the Rosenberg (1979) self-esteem scale on a 1 (Strongly disagree) to 7 (Strongly agree) scale; $\alpha = .94$. If weakly identified minorities reported higher self-esteem in the multicultural company, this would be consistent with the positive self-view alternative explanation (and inconsistent with the self-stereotyping hypothesis).

Racial identification. Participants responded to the same four items used in previous experiments in both Wave 1 ($\alpha = .83$) and Wave 2 ($\alpha = .82$), and they were strongly associated across waves, $r(350) = .68$, $p < .001$. Additionally, racial identification did not significantly change between Wave 1 ($M = 4.84$, $SD = 1.57$) and Wave 2 ($M = 4.75$, $SD = 1.61$), $t(351) = 1.32$, $p = .187$, $d = 0.07$. Importantly, participants' level of racial identification did not differ across conditions in Wave 1, $F(2, 349) = 1.07$, $p = .344$, suggesting successful random assignment, or in Wave 2, $F(2, 349) = 1.03$, $p = .359$. For the main analyses, we used the Wave 1 measure of racial identification.

Table S7

Means, Standard Deviations, and Correlations Between Primary Experiment 3 Variables

	1	2	3	4	5	6	7	8	9
1. Racial Identification	-								
2. Black Activity Stereotypes	.16**	-							
3. Positive Black Trait Stereotypes	.08	.72***	-						
4. Interest in Black Network	.38***	.26***	.16**	-					
5. Authenticity	.16**	.33***	.33***	.29***	-				
6. Anxiety	-.01	-.23***	-.23***	-.01	-.46***	-			
7. Race Concerns	.18***	-.13*	-.13*	.08	-.36***	.45***	-		
8. Workplace Citizenship	.09	.26***	.36***	.32***	.40***	-.17**	-.11*	-	
9. Self-Esteem	.05	.23***	.34***	.22***	.55***	-.39***	-.33***	.38***	-
Mean (*SD*)	4.84 (1.57)	4.02 (1.06)	4.31 (1.15)	3.88 (1.24)	5.73 (1.21)	3.67 (1.65)	3.19 (1.78)	3.70 (0.86)	5.70 (1.34)

Note. *N*s range from 350 to 352. Numbers in parentheses next to means correspond to standard deviations. Scales range from 1-7 for all measures except Interest in Black Network, which ranges from 1-5.

* $p < .05$ ** $p < .01$ *** $p < .001$

Results

Manipulation checks. Perceptions of how much the company focused on group differences differed by condition, $F(2,346) = 53.95$, $p < .001$. Specifically, Bonferroni multiple comparison tests revealed that participants perceived greater focus on group differences in the multiculturalism condition ($M = 6.35$, $SD = 1.02$) than in the colorblind condition ($M = 4.27$, $SD = 2.24$), $p < .001$, and the control condition ($M = 4.73$, $SD = 1.39$), $p < .001$. However, participants did not perceive a difference in how much the control and colorblind companies focused on group differences, $p = .092$.

Main analyses

Authenticity. Contrary to Experiment 1, there was no interaction between racial identification and diversity condition, *p*s > .40 (see Table S8). However, a main effect showed that participants would feel more comfortable being authentic when exposed to a company that valued multiculturalism ($M = 6.05$) relative to colorblindness ($M = 5.54$) and the control company ($M = 5.58$).

Anxiety. Contrary to Experiment 1, there was no interaction between racial identification and diversity condition, *p*s > .112 (see Table S8), although the pattern of interaction results was consistent with Experiment 1. A main effect showed that participants would feel less anxious when exposed to a company that valued multiculturalism relative to colorblindness, but the ΔR^2 for Step 1 was not statistically significant.

Race concerns. Contrary to hypotheses, there was no interaction between racial identification and diversity condition, *p*s > .560 (see Table S8).

Table S8

Hierarchical Regression on Authenticity, Anxiety, and Race Concerns in Experiment 3

	Authenticity		Anxiety		Race Concerns	
Predictor	β	*p*	β	*p*	β	*p*
Step 1	$\Delta R^2 = 0.06, p < .001$		$\Delta R^2 = 0.01, p = .265$		$\Delta R^2 = 0.03, p = .009$	
Racial Identification	0.15	.004	-0.003	.951	0.18	< .001
Control (v. Multicultural)	-0.18	.002	0.06	.307	0.01	.858
Colorblind (v. Multicultural)	-0.19	.002	0.12	.047	-0.01	.856
Step 2	$\Delta R^2 = 0.002, p = .701$		$\Delta R^2 = 0.01, p = .273$		$\Delta R^2 = 0.001, p = .842$	
Control (v. Multicultural) x Identification	0.07	.402	0.05	.533	0.03	.727
Colorblind (v. Multicultural) x Identification	0.04	.613	0.12	.112	0.04	.560

Note. Regression coefficients are reported from the step on which each variable was first entered. The multicultural condition, the reference group in the regression, is always coded as 0, with control and colorblindness coded as 1.

Self-stereotyping. Our analytic strategy and hypotheses were identical to Experiments 1 and 2, but used the measure of racial identification from Wave 1. Contrary to Experiment 2, there were no main effects or interactions with diversity condition on any of the three self-stereotyping measures (statistics reported in the main text). Because interest in the CCG Network was left-skewed (42% of people selected the highest option, “Extremely interested“), we also examined this variable as a binary outcome (1 = “Extremely interested”, 0 = All options below “Extremely interested”) in a logistic regression. However, there were still no main effects or interactions, *p*s > .385.

Workplace citizenship. To assess whether participants self-presented more positively in general (not just on stereotype-relevant traits), we assessed reports of their past work histories. If weakly identified minorities described their work histories more positively in the multicultural company, this would be consistent with the positive self-presentation alternative explanation (and inconsistent with hypotheses). This measure was less relevant given that we did not find an effect on self-stereotyping in this study, and indeed, there was no effect of condition or interaction with condition and racial identification, *p*s > .452.

State self-esteem. We assessed trait self-esteem for similar reasons as workplace citizenship, but there was no effect of condition or interaction with condition and racial identification, *p*s > .147.

Full Details of Experiment 4

This experiment was pre-registered at https://osf.io/sahtb.

Method

Participants. Of 615 African American Project Implicit participants (previous participants were not permitted to participate), 14 were excluded because 10% or more of their IAT trials were faster than 300 milliseconds. Of the remaining 601, 368 reached the end (246 women, 121 men, 1 unspecified; mean age = 34.13, *SD* = 12.88; 85% had completed

some college or a higher level of education). We used G*power 3.1.5 to estimate the goal sample size needed to detect an R^2 change of .023 (effect size estimated from Study 2) when adding an interaction into a linear bivariate regression with 80% power, an alpha level of 0.05. Based on this, we anticipated needing 336 participants – to account for potential exclusions, we collected data until we reached 375 participants (see pre-registration details at https://osf.io/5nvwc/)

Procedure. Participants were randomly assigned to read the same multiculturalism or colorblind philosophy in a CCG recruitment brochure from previous experiments or a control statement that described CCG's staff philosophy without any reference to diversity. They next imagined that they were interviewing at CCG and completed the activity self-stereotyping measure (rap music, gospel music, talking/socializing, sports/fitness, athletics, track, physical education, basketball, attending religious services, singing, activities related to music; α = .72).

Next, participants responded to measures assessing workplace citizenship (α = .74), interest in a race-relevant sub-organization, anticipated authenticity (α = .85) and anxiety (α = .79) in the interview, race concerns (ρ = .76), and racial identification (α = .75), in that order. Participants' level of racial identification did not differ across conditions, $F(2, 365) = 1.02$, $p = .363$. Finally, participants completed the same manipulation check item as in Experiment 2 and an IAT.

Table S9

Means, Standard Deviations, and Correlations Between Primary Experiment 4 Variables

	1	2	3	4	5	6	7
1. Racial Identification	-						
2. Black Activity Stereotypes	.06	-					
3. Interest in Black Network	.29***	.11*	-				
4. Authenticity	-.04	.10*	.15**	-			
5. Anxiety	-.05	-.13**	-.11*	-.42***	-		
6. Race Concerns	.16**	-.09	-.06	-.29***	.43***	-	
7. Workplace Citizenship	.15 **	.13**	.16**	.17***	-.18***	-.12*	-
Mean (*SD*)	4.87 (1.57)	4.60 (0.98)	5.90 (1.42)	5.57 (1.31)	3.82 (1.51)	3.50 (1.83)	4.30 (0.90)

Note. *N*s for correlations range from 364 to 370. Numbers in parentheses next to means correspond to standard deviations. Scales range from 1-7 for all measures except Interest in Black Network, which ranges from 1-5.

$p < .05$ ** $p < .01$ *** $p < .001$

Results

Manipulation and attrition checks. Perceptions of how much the company focused on group differences differed by condition, $F(2, 336) = 15.36$, $p < .001$. Specifically, Bonferroni multiple comparison tests revealed that participants perceived greater focus on group differences in the multiculturalism condition ($M = 5.37$, $SD = 1.97$) than in the colorblind condition ($M = 3.94$, $SD = 2.30$), $p < .001$, and the control condition ($M = 4.32$, $SD = 1.83$), $p = .001$. However, participants did not perceive a difference in how much the control and colorblind companies focused on group differences, $p = .363$. Attrition from the study did not differ by condition, $\chi^2(2, n = 601) = 2.93$, $p = .231$, or gender, $\chi^2(1, n = 599) = 0.24$, $p = .621$.

Analytic strategy. Our analytic strategy and hypotheses were the same as in previous experiments.

Authenticity. The predicted two-way interaction emerged between the multiculturalism condition (relative to colorblindness) and racial identification (see Table S10 for statistics), but not between the multiculturalism condition (relative to control) and racial identification.[5] Next, we examined simple effects for both the multicultural relative to colorblind and the multicultural relative to control comparisons.

In simple effects analyses, consistent with predictions, African Americans with weak racial identification reported that they would feel less comfortable being authentic when exposed to a company that valued multiculturalism relative to colorblindness, $b = 0.71$, $SE =$

[5] We also re-ran the regression model with the control condition as the reference group to compare the control and colorblind conditions. There was a significant interaction between the colorblind condition (relative to control) and racial identification, $\beta = -0.20$, $t(362) = -2.55$, $p = .011$. African Americans with weak racial identification reported that they would feel less comfortable being authentic when exposed to a control company than to one that valued colorblindness, $b = 1.11$, $SE = 0.35$, $p = .002$. This was not the case for participants stronger in racial identification, $b = 0.03$, $SE = 0.20$, $p = .869$.

0.33, *p* = .032. African Americans with strong racial identification showed the opposite pattern, but this difference did not reach conventional levels of statistical significance, *b* = -0.24, *SE* = 0.20, *p* = .247.

Simple slope analyses partially confirmed the predicted interaction pattern as well. As participants in the colorblind condition were more strongly racially identified, they felt less comfortable being authentic, *b* = -0.18, *SE* = 0.07, *p* = .009. Although we predicted a positive relationship between racial identification and authenticity in the multicultural condition, it was not statistically significant for either the multicultural or control conditions, *b* = 0.06, *SE* = 0.08, *p* = .464, *b* = 0.09, *SE* = 0.08, *p* = .275, respectively.

Anxiety. The predicted two-way interaction emerged between the multiculturalism condition (relative to colorblindness) and racial identification, but not between the multiculturalism condition (relative to control) and racial identification.[6] Next, we examined simple effects for both the multicultural relative to colorblind and the multicultural relative to control comparisons.

In simple effects analyses, consistent with predictions, African Americans with weak racial identification reported that they would feel more anxious when exposed to a company that valued multiculturalism relative to colorblindness, *b* = -1.12, *SE* = 0.37, *p* = .003. However, African Americans with strong racial identification reported that they would feel less anxious when exposed to a company that valued multiculturalism relative to

[6] We also re-ran the regression model with the control condition as the reference group to compare the control and colorblind conditions. There was a significant interaction between the colorblind condition (relative to control) and racial identification, β = 0.31, *t*(362) = 3.94, *p* < .001. African Americans with weak racial identification reported that they would feel more anxious when exposed to a control company than to one that valued colorblindness, *b* = -1.88, *SE* = 0.40, *p* < .001. This was not the case for participants stronger in racial identification, *b* = -0.01, *SE* = 0.23, *p* = .971.

colorblindness, *b* = 0.31, *SE* = 0.23, *p* = .187 (although it was only significant when using the Johnson-Neyman technique, presented in Table 4).

Simple slope analyses partially confirmed the predicted interaction pattern. As participants in the colorblind condition were more strongly racially identified, they felt more anxious, *b* = 0.19, *SE* = 0.08, *p* = .014. In the multiculturalism condition, this relationship was attenuated, and in the negative direction but it did not reach conventional levels of statistical significance, *b* = -0.16, *SE* = 0.09, *p* = .063. Although we predicted a null relationship between racial identification and anxiety in the control condition, the relationship was significantly negative, *b* = -0.27, *SE* = 0.09, *p* = .002, showing that as participants were more strongly racially identified, they felt less anxious in a neutral workplace environment. Taken together, our predictions were confirmed for the colorblind and multicultural conditions, but not for the control condition.

Race concerns. Contrary to hypotheses, there was no interaction between racial identification and diversity condition. However, there was an unexpected main effect such that participants in the multicultural condition (*M* = 3.24) reported fewer race concerns than those in the control condition (*M* = 3.78), but not relative to the colorblind condition (*M* = 3.51). Although originally unexpected, these results partially conceptually replicate findings by Purdie-Vaughns, Steele, Davies, Ditlmann, and Crosby (2008) suggesting that racial minorities are more likely to trust multicultural companies.

Table S10

Hierarchical Regression on Authenticity, Anxiety, and Race Concerns in Experiment 4

	Authenticity		Anxiety		Race Concerns	
Predictor	β	*p*	β	*p*	β	*p*
Step 1	$\Delta R^2 = 0.01$, $p = .206$		$\Delta R^2 = 0.02$, $p = .041$		$\Delta R^2 = 0.04$, $p = .003$	
Racial Identification	-0.03	.520	-0.06	.263	0.16	.003
Control (v. Multicultural)	-0.10	.105	0.12	.043	0.12	.039
Colorblind (v. Multicultural)	0.01	.812	-0.03	.583	0.06	.296
Step 2	$\Delta R^2 = 0.02$, $p = .017$		$\Delta R^2 = 0.05$, $p < .001$		$\Delta R^2 = 0.003$, $p = .592$	
Control (v. Multicultural) x Identification	0.02	.783	-0.06	.369	0.07	.336
Colorblind (v. Multicultural) x Identification	-0.18	.022	0.23	.002	0.02	.842

Note. Regression coefficients are reported from the step on which each variable was first entered. The multicultural condition, the reference group in the regression, is always coded as 0, with control and colorblindness coded as 1.

Secondary analyses

African American activity stereotypes. Contrary to hypotheses, there were no interactions with diversity condition, *p*s > .523, ΔR^2 = .001, *p* = .767. However, there was an unexpected main effect such that participants in the colorblind condition (*M* = 4.78) described themselves as more stereotypical than those in the multicultural condition (*M* = 4.53), β = 0.12, *t*(364) = 2.09, *p* = .037. This pattern of results did not match hypotheses or the findings in any previous experiments, so may not be reliable.

Interest in Black CCG Network. Contrary to hypotheses, there were no interactions with diversity condition, *p*s > .081, ΔR^2 = .01, *p* = .218. However, there was an unexpected main effect such that participants in the colorblind condition (*M* = 6.08) reported more interest in the Black CCG Network than those in the multicultural condition (*M* = 5.68), β = 0.12, *t*(358) = 2.03, *p* = .043. Because interest in the CCG Network was left-skewed, we also examined this variable as a binary outcome (1 = "Extremely interested", 0 = All options below "Extremely interested") in a logistic regression. In this analysis, there were no main effects of condition or interactions, *p*s > .378. This pattern of results did not match hypotheses or the findings from any previous experiments.

Workplace citizenship. To assess whether participants self-presented more positively in general (not just on stereotype-relevant traits), we assessed reports of their past workplace citizenship. If weakly identified minorities described their work histories more positively in the multicultural company, this would be consistent with the positive self-presentation alternative explanation (and inconsistent with hypotheses).

This measure was less relevant given that we did not find an effect on self-stereotyping; however, a two-way interaction (ΔR^2 = .02, *p* = .036) emerged between the multiculturalism condition (relative to colorblindness) and racial identification, β = -0.17, *t*(361) = -2.20, *p* = .028, but not for the multiculturalism condition (relative to control) and

racial identification, β = 0.002, *t*(361) = 0.03, *p* = .976. Next, we examined simple effects for the multicultural relative to colorblind comparison.

In simple effects analyses, inconsistent with the alternative explanation, African Americans with weak racial identification presented themselves less positively when exposed to a company that valued multiculturalism relative to colorblindness, *b* = 0.52, *SE* = 0.23, *p* = .023. This finding may be another manifestation of the increased anxiety reported by weakly identified participants considering the multicultural context. African Americans with strong racial identification did not show a significant difference by condition, *b* = -0.10, *SE* = 0.14, *p* = .461.

Implicit self-stereotyping. There were no effects of diversity condition on implicit self-stereotyping, *p*s > .106.

Full Details of Experiment 5

Method

Participants. Of 386 African American Project Implicit participants (previous participants were not permitted to participate), eleven were excluded because 10% or more of their BIAT trials were faster than 300 milliseconds, and one was excluded for not taking the essay task seriously (as determined by four coders). Of the remaining 374, 204 reached the end of the study (146 women, 56 men, 2 unknown; mean age = 34.98, *SD* = 13.85; 95% had completed some college or a higher level of education). Accounting for attrition, this left adequate power (π = .80) to detect a slope difference by condition (i.e., an interaction between racial identification and condition) of β = 0.21 for authenticity. Our goal sample size was 70 per condition.

Additional information about primary measures. Participants imagined that they were interviewing at CCG and completed the positive trait measures from Experiment 2, with the trait religious added to the African American stereotypes to boost reliability. Participants

indicated the extent to which positive stereotypes of African Americans (streetwise, humorous, athletic, musical, emotionally expressive, and religious; $\alpha = .54$) were self-descriptive. Participants did not self-report about negative traits or activity interests in this study. As in previous experiments, participants' level of racial identification ($\alpha = .69$) did not differ across conditions, $F(2, 204) = 2.22$, $p = .111$.

Table S11

Means, Standard Deviations, and Correlations Between Primary Experiment 5 Variables

	1	2	3	4	5
1. Racial Identification	-				
2. Positive Trait Stereotypes (self-report)	.09	-			
3. Authenticity (essay)	.12	-.05	-		
4. Anxiety (essay)	.01	-.10	-.51***	-	
5. Hiring Desirability	.25**	-.12*	.45***	-.44***	-
Mean (*SD*)	4.42 (1.47)	4.34 (1.11)	4.71 (0.61)	1.75 (0.54)	3.71 (0.80)

Note. Ns range from 175 to 204. Numbers in parentheses next to means correspond to standard deviations. Scales range from 1-7 for all measures.

* $p < .05$ ** $p < .01$ *** $p < .001$

Supplementary dependent measures

Positive self-presentation essay coding. If there were differences in hiring outcomes across diversity conditions, we hypothesized that this would be because different levels of anxiety and inauthenticity were evident in their self-descriptions. Specifically, we expected that in the multicultural condition, weakly identified participants would be seen as less desirable applicants because their anxiety and inauthenticity would be more evident in their self-descriptions than those in the colorblind condition (and the reverse for strongly identified participants).

However, an alternative possibility was that in the multicultural condition, weakly identified participants would be seen as less desirable applicants because they were less interested in working at the organization than those in the colorblind condition – this might lead them to present themselves less positively in reaction (and the reverse for strongly identified participants).

To address positive self-presentation as an alternative explanation for differences in hiring outcomes, we asked three research assistants to code the essays for positive self-presentation on a 1 (Not at all) to 5 (Extremely) scale: "How competent does this person seem?" and "Does this person convey a positive impression?" Coders were blind to all hypotheses and experimental condition of the essay writer, and their responses, which had moderate interrater reliability (*ICC* = .63), were averaged to create a measure of positive self-presentation for each essay.

Desire to work at company and hiring perceptions. The primary purpose of the research was to understand how diversity approaches shape self-views in the organizational context. However, we also directly assessed participants' perception of the company because it might have been affected by anticipated feelings of authenticity and anxiety. Desire to work

at the company was measured with "How much would you want to work at CCG?" on a 1 (Not at all) to 7 (Very much so) scale, and perception that the company would hire participants was measured with "How likely would CCG be to hire you?" on a 1 (Not at all likely) to 7 (Very likely) scale. We did not include these measures in the main results for the sake of brevity, because it was only measured once, and because it contributed less to understanding the phenomenon at hand than did the measures included in the main text.

African American similarity. African American similarity consisted of three items ("I am similar to other African Americans in terms of my behaviors"; "I am similar to the average African American"; "I am similar to other African Americans in terms of my life goals"; α = .81) on a 1 (Strongly Disagree) to 7 (Strongly Agree) scale and was measured immediately before racial identification and after the CCG interview questions. We included this measure of ingroup similarity as another way of assessing whether diversity approaches affected how people viewed or presented themselves. We did not include this measure in the main results for the sake of brevity, because it was only measured once, and because it showed null results parallel to the self-stereotyping measure presented in the main text (i.e., it was redundant information).

Private collective self-esteem. Private collective self-esteem (Luhtanen & Crocker, 1992) was measured with four items (α = .75; e.g., "I feel good about the race/ethnicity I belong to") using the same scale. We included this measure to understand whether diversity approaches affect how participants feel about their group identity. If weakly identified participants self-stereotyped more in the multicultural relative to colorblind condition (as originally hypothesized), one possibility would be that it made them feel more positive toward their own group and therefore more comfortable embracing stereotypical qualities.

Results

Manipulation and attrition checks. Perceptions of how much the company focused on group differences differed by condition, $F(2, 186) = 28.19$, $p < .001$. Specifically, Bonferroni multiple comparison tests revealed that participants perceived greater focus on group differences more in the multiculturalism condition ($M = 3.31$, $SD = 1.04$) than in the colorblind condition ($M = 1.94$, $SD = 1.27$), $p < .001$, and the control condition ($M = 2.19$, $SD = 1.06$), $p < .001$. However, participants did not perceive a difference in how much the control and colorblind companies focused on group differences, $p = .642$. Attrition from the study did not differ by condition, $\chi^2(2, n = 374) = 2.74$, $p = .255$, or gender, $\chi^2(1, n = 371) = 1.88$, $p = .170$.

Primary dependent measures. Our analytic strategy and hypotheses were similar to previous experiments.

Authenticity. The predicted two-way interaction emerged between the multiculturalism condition (relative to colorblindness) and racial identification, but not for the multiculturalism condition (relative to control) and racial identification (see Table S12).[7] Next, we examined simple effects for the multicultural relative to colorblind comparisons.

In simple effects analyses, African Americans with weak racial identification seemed less authentic when exposed to a company that valued multiculturalism relative to colorblindness, but this difference was not statistically significant, $b = 0.29$, $SE = 0.21$, $p = .171$. African Americans with strong racial identification showed the opposite significant pattern, $b = -.36$, $SE = .17$, $p = .032$.

Simple slope analyses partially confirmed the predicted interaction pattern. Although we predicted a negative relationship between racial identification and authenticity in the

[7] We also re-ran the regression model on authenticity with the control condition as the reference group to compare the control and colorblind conditions. There was no interaction between the colorblind condition (relative to control) and racial identification, $\beta = -0.04$, $t(169) = -0.33$, $p = .742$.

colorblind condition, this slope was not statistically significant, *b* = -0.02, *SE* = 0.05, *p* = .761. However, as predicted in the multiculturalism condition, as participants were more strongly racially identified, they seemed more authentic, *b* = 0.15, *SE* = 0.05, *p* = .008. Finally, as expected, the relationship between racial identification and authenticity was not statistically significant in the control condition, *b* = 0.01, *SE* = 0.06, *p* = .869.

Anxiety. The predicted two-way interaction emerged between the multiculturalism condition (relative to control) and racial identification (see Table S12), but was not statistically significant for multiculturalism relative to colorblindness, *p* = .061.[8] Nonetheless, we examined simple effects for both the multicultural relative to colorblind and the multicultural relative to control comparisons to understand the pattern of effects.

In simple effects analyses, consistent with predictions, African Americans with weak racial identification showed more anxiety when exposed to a company that valued multiculturalism relative to control, *b* = -0.51, *SE* = 0.21, *p* = .017, and colorblindness, but it was not statistically significant for the latter comparison, *b* = -0.33, *SE* = 0.19, *p* = .084. African Americans with strong racial identification showed the opposite pattern, but these differences were not statistically significant for the multicultural relative to control comparison, *b* = 0.21, *SE* = 0.14, *p* = .135, or the multicultural relative to colorblind comparison, *b* = 0.19, *SE* = 0.15, *p* = .224.

Simple slope analyses partially confirmed the predicted interaction pattern. Although we predicted a positive relationship between racial identification and anxiety in the colorblind condition, this slope was not statistically significant, *b* = 0.03, *SE* = 0.05, *p* = .531. However, as predicted in the multiculturalism condition, as participants were more strongly racially

---

[8] We also re-ran the regression model on anxiety with the control condition as the reference group to compare the control and colorblind conditions. There was no interaction between the colorblind condition (relative to control) and racial identification, β = -.07, *t*(169) = -0.62, *p* = .534.

identified, they showed less anxiety, *b* = -0.10, *SE* = 0.05, *p* = .046. Finally, as expected, the relationship between racial identification and anxiety was not statistically significant in the control condition, *b* = 0.08, *SE* = 0.05, *p* = .119.

Positive African American traits. The predicted two-way interaction emerged between the multiculturalism condition (relative to colorblindness) and racial identification. However, the predicted two-way interaction between the multiculturalism condition (relative to control) and racial identification did not emerge. Because of this, we probed the multicultural relative to colorblind comparison, but not the multicultural relative to control comparison.

In simple effects analyses, consistent with predictions, African Americans with weak racial identification reported that stereotypically African American traits were more self-descriptive when exposed to a company that valued multiculturalism relative to colorblindness, *b* = -1.09, *SE* = 0.35, *p* = .002. African Americans with strong racial identification showed the opposite pattern, but this difference did not reach conventional levels of statistical significance, *b* = 0.45, *SE* = 0.28, *p* = .112.

Simple slope analyses confirmed the predicted interaction pattern as well. As participants in the colorblind condition were more strongly racially identified, they described themselves more stereotypically, *b* = 0.27, *SE* = 0.09, *p* = .003. Racial identification was unrelated to how stereotypically participants described themselves in the multiculturalism condition, *b* = -0.11, *SE* = 0.09, *p* = .200, and control condition, *b* = 0.03, *SE* = 0.09, *p* = .757.

Table S12

Hierarchical Regression on Primary Dependent Measures in Experiment 5

	Positive African American Self-Stereotyping		Essay Authenticity		Essay Anxiety	
Predictor	β	*p*	β	*p*	β	*p*
Step 1	ΔR^2 = .02, p = .388		ΔR^2 = .03, p = .156		ΔR^2 = .001, p = .983	
Racial Identification	0.08	.263	0.11	.152	0.01	.917
Control (v. Multicultural)	-0.08	.301	-0.14	.104	-0.03	.721
Colorblind (v. Multicultural)	-0.08	.321	-0.08	.348	-0.002	.984
Step 2	ΔR^2 = .04, p = .011		ΔR^2 = .03, p = .075		ΔR^2 = .04, p = .034	
Control (v. Multicultural) x Identification	0.11	.270	-0.19	.079	0.27	.012
Colorblind (v. Multicultural) x Identification	0.30	.003	-0.24	.033	0.21	.061

Note. Regression coefficients are reported from the step on which each variable was first entered. The multicultural condition, the reference group in the regression, is always coded as 0, with control and colorblindness coded as 1.

Positive self-presentation. We expected that differences in hiring desirability across diversity conditions was due to raters detecting anxiety and inauthenticity in participants' self-descriptions. However, another possibility was that diversity condition impacted interest in working at that company, and therefore efforts to present themselves positively. When examining positive self-presentation in essays as an alternative explanation for differences in hiring outcomes, there were no main effects or interactions between diversity condition and racial identification (see Table 3). This suggests that the interaction between diversity condition and racial identification on hiring desirability could not be attributed to participants changing their self-presentations due to more or less interest in a company context.

Implicit self-stereotyping. There were no effects of diversity condition on implicit self-stereotyping, *p*s > .536.

Authenticity and anxiety mediating hiring desirability. We tested moderated mediation models with 10,000 bootstrap resamples using the PROCESS macro (Model 8; Hayes, 2013) to determine whether authenticity and anxiety statistically mediated the interaction effect of multiculturalism (relative to colorblindness) on hiring desirability. The index of moderated mediation was significant for authenticity, $b = -0.07$, $SE = 0.04$, 95% *CI* [-0.18, -0.01], but not anxiety, $b = -0.05$, $SE = 0.03$, 95% *CI* [-0.13, 0.003]. When examining mediation by authenticity at different levels of the racial identification moderator, there was only a significant indirect effect among participants who were strongly identified, $b = -0.15$, $SE = 0.10$, 95% CI[-0.38, -0.01]. Taken together, this suggests that increased feelings of authenticity in the multicultural relative to colorblind condition mediated better hiring outcomes among those strongly identified.

Authenticity and anxiety mediating self-stereotyping. Using the same model as above, the index of moderated mediation was not significant for anxiety, *b* = -0.05, *SE* = 0.04, 95% CI[-0.14, 0.006], or authenticity, *b* = 0.02, *SE* = 0.04, 95% CI[-0.05, 0.11].

Supplementary dependent measures

There were no significant effects of diversity condition or interaction between diversity condition and racial identification on participants' perception that they will be hired or their reported similarity to other African Americans (see Table S13).

Private collective self-esteem. As shown in Table S13, there was a significant interaction between colorblindness (v. multiculturalism) and racial identification on private collective self-esteem. However, it revealed that weakly racially identified participants had more positive feelings toward their racial group at the multicultural than the colorblind company, *b* = -0.68, *SE* = 0.34, *p* = .045. Strongly identified participants were unaffected, *b* = 0.35, *SE* = 0.27, *p* = .188. Although the finding among weakly identified participants should be interpreted cautiously without replication, it is intriguing – it suggests that multiculturalism might make weakly identified minorities have more positive attitudes toward their group identity, while simultaneously feeling uncomfortable with the focus on that group identity. This ambivalence may be specific to a work context in which they prefer a focus on the individual self rather than group identity.

Desire to work at company. A significant interaction between diversity condition and racial identification revealed that *strongly* racially identified participants wanted to work at

the multicultural more than the colorblind, *b* = -1.56, *SE* = 0.54, *p* = .004, or control companies, *b* = -2.02, *SE* = 0.52, *p* < .001. The opposite was true for weakly identified participants, but it did not reach conventional levels of statistical significance, *p*s > .083.

Table S13

Hierarchical Regression on Supplemental Dependent Variables in Experiment 5

	African American Similarity		Private Collective Self-Esteem		Desire to Work at Company		Perceptions of Being Hired	
Predictor	β	*p*	β	*p*	β	*p*	β	*p*
Step 1	$\Delta R^2 = 0.16$, $p < .001$		$\Delta R^2 = 0.10$, $p < .001$		$\Delta R^2 = 0.05$, $p = .028$		$\Delta R^2 = 0.02$, $p = .250$	
Racial Identification	0.38	<.001	0.30	<.001	-0.09	.388	-0.06	.424
Control (v. Multicultural)	0.02	.839	0.07	.320	-0.24	.003	-0.15	.068
Colorblind (v. Multicultural)	-0.05	.528	-0.04	.599	-0.11	.177	-0.04	.629
Step 2	$\Delta R^2 < 0.001$, $p = .993$		$\Delta R^2 = 0.03$, $p = .042$		$\Delta R^2 = 0.05$, $p = .009$		$\Delta R^2 = 0.02$, $p = .125$	
Control (v. Multicultural) x Identification	-0.004	.962	-0.02	.860	-0.25	.011	-0.20	.044
Colorblind (v. Multicultural) x Identification	0.01	.942	0.20	.034	-0.28	.006	-0.08	.455

Note. Regression coefficients are reported from the step on which each variable was first entered. The multicultural condition, the reference group in the regression, is always coded as 0, with control and colorblindness coded as 1.

Stereotype Pilot Studies

Activity Stereotyping Pilot Study

With an independent sample of 13 University of Washington students (9 White, 3 Asian, 2 Hispanic), we pre-tested the 35 activities, interests, and traits from Steele and Aronson's (1995) stereotype avoidance measure to assess the stereotypicality of each activity for our activity self-stereotyping measure. Pre-test participants circled activities that they considered consistent with the cultural stereotype of African Americans (circled = 1, not circled = 0), and an activity was considered stereotypical if it was chosen at a rate significantly above chance (0.50). On this basis, nine activities were selected as stereotypical of African Americans (rap/hip-hop, football, sports, basketball, talking, gospel music, physical education, athletics, track) – we omitted the traits included in this scale because a separate measure assessed trait stereotypes (see next section). Means, standard deviations, and *p*-values indicating each item’s difference from 0.50 are reported in Table S14.

Table S14

Means and Standard Deviations of Activity Stereotypicality Pilot Ratings

Activity	Mean	*SD*	*p*	Activity	Mean	*SD*	*p*
Athletics career*	1.00	0.00	-	Aloof	0.08	0.28	<.001
Football*	1.00	0.00	-	Educator as career	0.08	0.28	<.001
Basketball*	0.92	0.28	<.001	Foreign language courses	0.08	0.28	<.001
Rap/Hip-hop*	0.92	0.28	<.001	Professional (doctor, lawyer) career	0.08	0.28	<.001
Sports*	0.92	0.28	<.001	Serious	0.08	0.28	<.001
Talking*	0.92	0.28	<.001	Soccer	0.08	0.28	<.001
Humorous	0.85	0.38	0.01	Anxious	0.00	-	-
Outgoing	0.85	0.38	0.01	Chess	0.00	-	-
Aggressive	0.77	0.44	0.05	Classical	0.00	-	-
Gospel music*	0.77	0.44	0.05	Community service	0.00	-	-
Physical education courses*	0.77	0.44	0.05	Country music	0.00	-	-
Track*	0.77	0.44	0.05	Fuzzy	0.00	-	-
Dancing	0.62	0.51	0.43	Golf	0.00	-	-
Active	0.54	0.52	0.79	Hockey	0.00	-	-
Boxing	0.54	0.52	0.79	Martial Arts	0.00	-	-
Business career	0.54	0.52	0.79	Math & science courses	0.00	-	-
Rhythm & Blues	0.54	0.52	0.79	New Age	0.00	-	-
Jazz	0.46	0.52	0.79	Organized	0.00	-	-
Happy	0.38	0.51	0.43	Reading	0.00	-	-
Lazy	0.31	0.48	0.17	Rock music	0.00	-	-
Shopping	0.31	0.48	0.17	Swimming	0.00	-	-
Being a lazy couch potato	0.23	0.44	0.05	Techie	0.00	-	-
Mild	0.15	0.38	0.01	Tennis	0.00	-	-
Verbal courses	0.15	0.38	0.01	Traveling	0.00	-	-
Warm	0.15	0.38	0.01				

Note. Responses were binary (selected as stereotypical or not). A one-sample t-test compared the mean response for each trait to chance (0.50).

* Items included in experiments

Trait Stereotype Testing

Although the trait self-stereotyping measure was comprised of validated scales used in past research (see Judd et al., 1995; Wolsko et al., 2000) and validated in more recent research (e.g., Czopp & Monteith, 2006; Ghavami & Peplau, 2012), we conducted our own pilot study to confirm the appropriateness of these traits and to ensure that they also accounted for stereotypes of African American women. With an independent sample of 122 University of Washington students (see Table S15 for racial breakdown), we pre-tested 23 positive African American traits from Wolsko, Park, Judd, and Wittenbrink's (2000) stereotyping measure.

Pre-test participants were asked to circle all of the traits (circled = 1, not circled = 0) that encompassed stereotypes of either (between-subjects) African Americans, African American men, or African American women. A trait was considered stereotypical of African Americans if it was significantly above chance (0.50) either for the group as a whole or specifically for African American men or women.

Table S15

Racial Background of Participants Completing Trait Stereotypicality Pilot Ratings

Stereotype Target Condition	Participant Race					
	White	Asian	Latinx	Multiracial	Other	Total
African Americans	31	0	1	3	2	37
African American men	39	2	0	2	1	44
African American women	33	2	0	3	3	41
Total	103	4	1	8	6	122

Trait means, standard deviations, and *p*-values are in Table S16 below. Final scales in Experiment 1 included five traits that were positive stereotypes of African Americans (streetwise, humorous[9], athletic, musical, emotionally expressive). In Experiment 5, we added the item religious to the positive African American stereotype measure to boost reliability.

[9] Humorous was included because it was considered stereotypical in the first stereotype pilot we reported above, based on Steele and Aronson's (1995) measure.

Table S16

Results for Trait Stereotypicality Pilot Ratings

Trait	Overall (*N* = 38)			Men (*N* = 44)			Women (*N* = 40)		
	Mean	*SD*	*p*	Mean	*SD*	*p*	Mean	*SD*	*p*
Athletic*	0.92	0.27	<.001	0.86	0.35	<.001	0.50	0.51	>.99
Streetwise*	0.89	0.31	<.001	0.80	0.41	<.001	0.85	0.36	<.001
Religious**	0.71	0.46	0.01	0.50	0.51	>.99	0.58	0.50	0.35
Musical*	0.66	0.48	0.05	0.47	0.50	0.65	0.33	0.47	0.02
Emotionally expressive*	0.53	0.51	0.75	0.25	0.44	<.001	0.83	0.38	0.00
Humorous*	0.45	0.50	0.52	0.43	0.50	0.37	0.30	0.46	<.001
Fashionable	0.32	0.47	0.02	0.32	0.47	0.01	0.38	0.49	0.11
Playful	0.26	0.45	<.001	0.23	0.42	<.001	0.23	0.42	<.001
Cheerful	0.21	0.41	<.001	0.18	0.39	<.001	0.25	0.44	<.001
Charming	0.13	0.34	<.001	0.39	0.49	0.13	0.05	0.22	<.001
Merry	0.05	0.23	<.001	0.07	0.25	<.001	0.05	0.22	<.001
Sensitive	0.00	-	-	0.05	0.21	<.001	0.10	0.30	<.001

Note. Responses were binary (selected as stereotypical or not). A one-sample t-test compared the mean response for each trait to chance (0.50).

* Items included in experiments

** Item added after Experiment 2

References

Amodio, D. M., & Devine, P. G. (2006). Stereotyping and evaluation in implicit race bias: evidence for independent constructs and unique effects on behavior. *Journal of Personality and Social Psychology, 91*(4), 652–661. https://doi.org/10.1037/0022-3514.91.4.652

Biernat, M., Vescio, T. K., & Green, M. L. (1996). Selective self-stereotyping. *Journal of Personality and Social Psychology*, *71*(6), 1194–1209.

Cvencek, D., Greenwald, A. G., Brown, A. S., Gray, N. S., & Snowden, R. J. (2010). Faking of the Implicit Association Test is statistically detectable and partly correctable. *Basic and Applied Social Psychology*, *32*(4), 302–314. https://doi.org/10.1080/01973533.2010.519236

Czopp, A. M., & Monteith, M. J. (2006). Thinking well of African Americans: Measuring complimentary stereotypes and negative prejudice. *Basic and Applied Social Psychology, 28*(3), 233–250.

Devine, P. G., & Elliot, A. J. (1995). Are racial stereotypes really fading? The Princeton trilogy revisited. *Personality and Social Psychology Bulletin*, *21*(11), 1139–1150.

Eisinga, R., Grotenhuis, M. Te, & Pelzer, B. (2013). The reliability of a two-item scale: Pearson, Cronbach, or Spearman-Brown? *International Journal of Public Health*, *58*(4), 637–642. https://doi.org/10.1007/s00038-012-0416-3

Faul, F., Erdfelder, E., Buchner, A., & Lang, A. (2009). Statistical power analyses using G*Power 3.1 : Tests for correlation and regression analyses. *Behavior Research Methods*, *41*(4), 1149–1160. https://doi.org/10.3758/BRM.41.4.1149

Gawronski, B., LeBel, E. P., & Peters, K. R. (2007). What do implicit measures tell us? Scrutinizing the validity of three common assumptions. *Perspectives on Psychological Science*, *2*(2), 181–193. https://doi.org/10.1111/j.1745-6916.2007.00036.x

Ghavami, N., & Peplau, L. A. (2012). An intersectional analysis of gender and ethnic stereotypes: Testing three hypotheses. *Psychology of Women Quarterly, 37*(1), 113–127. https://doi.org/10.1177/0361684312464203

Greenwald, A., Nosek, B., & Banaji, M. (2003). Understanding and using the Implicit Association Test: I. An improved scoring algorithm. *Journal of Personality and Social Psychology, 85*(2), 197–216. https://doi.org/10.1037/0022-3514.85.2.197

Hayes, A. F. (2013). *Introduction to Mediation, Moderation, and Conditional Process Analysis*. Guilford Press.

Judd, C. M., Park, B., Ryan, C. S., Brauer, M., & Kraus, S. (1995). Stereotypes and ethnocentrism: Diverging interethnic perceptions of African American and White American youth. *Journal of Personality and Social Psychology, 69*(3), 460–481.

https://doi.org/10.1037/0022-3514.69.3.460

Litman, L., Robinson, J., & Abberbock, T. (2017). TurkPrime.com: A versatile crowdsourcing data acquisition platform for the behavioral sciences. *Behavior Research Methods*, *49*(2), 433–442. https://doi.org/10.3758/s13428-016-0727-z

Luhtanen, R., & Crocker, J. (1992). A collective self-esteem scale: Self-evaluation of one's social identity. *Personality and Social Psychology Bulletin*, *18*(3), 302–318. https://doi.org/10.1177/0146167292183006

Purdie-Vaughns, V., & Eibach, R. P. (2008). Intersectional invisibility: The distinctive advantages and disadvantages of multiple subordinate-group identities. *Sex Roles*, *59*(5–6), 377–391. https://doi.org/10.1007/s11199-008-9424-4

Purdie-Vaughns, V., Steele, C. M., Davies, P. G., Ditlmann, R., & Crosby, J. R. (2008). Social identity contingencies: How diversity cues signal threat or safety for African Americans in mainstream institutions. *Journal of Personality and Social Psychology*, *94*(4), 615–630. https://doi.org/10.1037/0022-3514.94.4.615

Rosenberg, M. (1979). *Conceiving the self*. New York: Basic Books.

Sellers, R., Shelton, J., Cooke, D., Chavous, T., Rowley, S., & Smith, M. (1998). A multidimensional model of racial identity: Assumptions, findings, and future directions. In R. Jones (Ed.), *African American identity development: Theory, research, and intervention* (pp. 275–302). Hampton, VA: Cobb and Henry.

Sesko, A. K., & Biernat, M. (2010). Prototypes of race and gender: The invisibility of Black women. *Journal of Experimental Social Psychology*, *46*(2), 356–360. https://doi.org/10.1016/j.jesp.2009.10.016

Steele, C. M., & Aronson, J. (1995). Stereotype threat and the intellectual test performance of African Americans. *Journal of Personality and Social Psychology*, *69*(5), 797–811. Retrieved from http://www.ncbi.nlm.nih.gov/pubmed/7473032

Wolsko, C., Park, B., Judd, C. M., & Wittenbrink, B. (2000). Framing interethnic ideology: Effects of multicultural and color-blind perspectives on judgments of groups and individuals. *Journal of Personality and Social Psychology*, *78*(4), 635–654. https://doi.org/10.1037/0022-3514.78.4.635
